# Leveraging ADMET Profiling, Network Pharmacology, and Molecular Docking to Evaluate the Repurposing of Product Nkabinde for COVID-19 Treatment

**DOI:** 10.3390/biomedicines14051022

**Published:** 2026-04-30

**Authors:** Samuel Chima Ugbaja, Siphathimandla Authority Nkabinde, Magugu Nkabinde, Nceba Gqaleni

**Affiliations:** 1 Traditional Medicine, School of Medicine, University of KwaZulu Natal, Durban 4000, South Africa; nceba5850@gmail.com (S.A.N.); magugun@webmail.com (M.N.); 2 African Health Research Institute (AHRI), 719 Umbilo Road, Durban 4000, South Africa

**Keywords:** Product Nkabinde, COVID-19, SARS-CoV-2, drug repurposing, polyherbal formulation, network pharmacology, molecular docking, host-directed therapy

## Abstract

**Background**: The coronavirus disease 2019 (COVID-19), caused by SARS-CoV-2, remains a significant threat to global health. This continued threat is due to the emergence of new variants, the immune system’s limited ability to respond, and the limited effectiveness of available treatments for all individuals. Therefore, leveraging drug repurposing, a fast and inexpensive way to find other drugs that have already been shown to be safe and efficacious, becomes useful. This study leverages ADMET profiling, network pharmacology, and molecular docking to evaluate the repurposing of Product Nkabinde for COVID-19 treatment. **Methods**: ADMET analysis involving the bioactive phytochemicals of PN was evaluated for pharmacokinetic appropriateness and drug-likeness. Using topological analysis, a network of protein–protein interactions was built to identify hub genes, and predicted compound targets were intersected with COVID-19-associated genes to find shared targets. Their biological importance was characterized using functional enrichment analysis. The binding affinities of PN phytochemicals against hub proteins and SARS-CoV-2 viral proteases (Mpro and PLpro) were assessed by molecular docking using AutoDock Vina. To confirm docking accuracy, co-crystallized ligands were redocked using Schrodinger 2022-1. The multi-target therapeutic potential of PN in COVID-19 was assessed using this integrative network pharmacology and molecular docking technique. **Results**: Molecular docking demonstrated that PN phytochemicals displayed robust and persistent binding affinities for both viral and host targets. Oleanolic acid showed the best affinity toward Mpro (−12.9 kcal/mol vs. −8.3 kcal/mol), while quercetin-3-O-β-D-(6′-galloyl)-glucopyranoside showed better binding to PLpro (−8.4 kcal/mol vs. −6.4 kcal/mol). Procyanidin B2 toward HCK (−10.5 vs. −7.9 kcal/mol), diosgenin toward EGFR (−9.4 vs. −8.4 kcal/mol), rutin toward SRC (−10.5 vs. −7.8 kcal/mol), and pimelea factor P2 toward PIK3R1 (−11.0 vs. −8.2 kcal/mol) all showed significantly higher affinities than their corresponding co-crystallized ligands. Furthermore, procyanidin B2 demonstrated consistent binding to STAT1 and STAT3, confirming its role in modulating immune signals. Most of the PN phytochemicals show advantageous pharmacokinetic properties, including elevated anticipated gastrointestinal absorption and adherence to Lipinski’s rule of five, signifying favorable oral bioavailability and drug-like properties. Moreover, PN exhibits a remarkable multi-target binding capacity against both SARS-CoV-2 proteases and key host signaling proteins involved in immune regulation and inflammatory responses, as determined by this integrative network pharmacology and molecular docking investigation. **Conclusions**: PN’s prospects as a host-directed, antiviral treatment for COVID-19 are demonstrated by its coordinated modulation of the PI3K/AKT, JAK–STAT, SRC-family kinase, EGFR, and SYK pathways. These results necessitate further experimental and clinical validation, providing a solid computational basis for repurposing PN in the treatment of COVID-19.

## 1. Introduction

In addition to acute morbidity and death, COVID-19 continues to pose a significant public health burden through recurring epidemic waves, persistent transmission in susceptible populations, and long-term post-acute consequences that put further strain on already overburdened health systems. The global therapeutic landscape remains uneven, particularly in low- and middle-income countries (LMICs), where supply chain limitations, delayed access to novel therapeutics, and affordability barriers can hinder timely treatment, despite vaccines and a few direct-acting antivirals reducing the severity of outcomes. Furthermore, SARS-CoV-2 remains susceptible to rapid evolutionary change, and antiviral pressure can select for variations with decreased drug susceptibility, particularly in immunocompromised hosts with prolonged infections. This underscores the importance of diverse, multifaceted therapeutic approaches [1]. Considering this, drug repurposing has become a practical public health tactic for quickening the discovery of treatments during epidemics. Compared to de novo drug discovery, repurposing reduces attrition risk and shortens development times by utilizing prior knowledge of safety, pharmacology, and manufacturing processes. These benefits were especially crucial during the COVID-19 pandemic, when the urgency of clinical need prompted a thorough evaluation of current antivirals, anti-inflammatory drugs, immunomodulators, and supportive medications across a range of diseases. Current evaluations of repurposing for COVID-19 highlight its benefits for rapid reaction, flexible trial design, and logical candidate prioritization, which, when backed by data, can be utilized at scale [2,3]. From a public health perspective, the benefits of repurposing extend beyond speed. Repurposing may enhance accessibility by identifying treatments with easier availability, lower prices, and feasible distribution in decentralized locations, which are crucial factors for bolstering outbreak response in LMICs. The importance of treatments that can alter host pathways linked to inflammation, endothelial damage, thrombosis, and immune exhaustion, as well as antiviral mechanisms, is highlighted by the growing understanding that severe COVID-19 is caused by both viral replication and dysregulated host immune responses. The exploration of candidates with coupled or complementary mechanisms is further motivated by lessons learned from the COVID-19 treatment era, which demonstrate that effective clinical management often requires matching therapies to the stage of the illness, including immunomodulation in severe disease and antiviral methods early on [1,4,5].

A patented African polyherbal compound called Product Nkabinde (PN) was initially developed for HIV treatment. Its applicability to COVID-19 repurposing is based on a fundamental public health principle: candidates that have previously demonstrated immune modulation and antiviral activity in rigorous experimental systems should be systematically reevaluated for new indications where comparable biological challenges, such as viral persistence, immune dysregulation, and inflammatory pathology, are present. Crucially, PN is being positioned as a contender for organized scientific evaluation under contemporary translational criteria rather than as a substitute for conventional COVID-19 treatment. This aligns with the WHO’s requests for reliable evidence supporting traditional products in infectious disease situations [6,7]. PN’s repurposing justification is based on an expanding body of experimental literature. PN exhibited strong in vitro anti-HIV-1 activity in a peer-reviewed study published in Frontiers in Pharmacology (2025) [8]. It demonstrated sustained reductions in p24 antigen levels in HIV-1-infected peripheral blood mononuclear cells (PBMCs) and high levels of inhibition in TZM-bl infection assays. It also demonstrated activity against both subtype B and subtype C strains, as well as compatibility with specific antiretrovirals [8]. From a public health perspective, this is significant because it demonstrates biologically relevant antiviral effects in primary immune cells under standardized experimental conditions, which are more indicative of in vivo immunobiology than those observed in immortalized cell lines alone. Moreover, the immunomodulatory effects of PN on human immune cells have also been experimentally tested. PN can affect immune response markers under controlled laboratory conditions, as reported by Setlhare et al. [9], which examined the effects of PN on cytokine and cellular biomarkers in PBMCs from healthy donors. In the case of COVID-19, where clinical severity is closely linked to maladaptive immune activation, compromised antiviral interferon responses, and inflammatory cascades that lead to lung damage and systemic consequences, such immune regulation is especially pertinent. Although it is impossible to assume that HIV and SARS-CoV-2 are mechanistically equivalent, the presence of experimentally demonstrated immunomodulatory activity lends credence to the theory that PN may interact with host pathways important to viral infections, which, more generally, is a necessary premise for host-directed repurposing strategies [9,10]. The need for treatments that are scalable, potentially affordable, and appropriate for various health system settings, particularly those where access to more recent antivirals may be restricted or delayed, strengthens the case for public health. By increasing the candidate pool and prioritizing interventions that may work through various routes, rather than relying solely on single-target mechanisms, repurposing techniques can help protect against the evolving threat of antiviral resistance and inconsistent medication efficacy [2].

Consequently, ADMET profiling, encompassing absorption, distribution, metabolism, excretion, and toxicity, is another crucial aspect of contemporary drug discovery, enabling early predictions of pharmacokinetic properties, bioavailability, and the safety of candidate compounds before clinical assessment, is important. ADMET evaluation identifies compounds with ideal oral absorption, metabolic stability, and low toxicity risk, therefore minimizing late-stage medication failure and expediting therapeutic development. This is especially crucial for natural compounds, which often exhibit intricate chemical structures and varied biological activities, yet require systematic pharmacokinetic assessment to determine translational significance. Recent advancements in computational ADMET methods, such as SwissADME and associated in silico platforms, have improved the effectiveness of screening phytochemicals for drug-likeness, facilitating the identification of potential repurposing candidates for emerging diseases such as COVID-19 [11,12,13]. In the context of SARS-CoV-2, ADMET profiling is essential for identifying orally bioavailable compounds that can attain therapeutic concentrations while ensuring acceptable safety and distribution profiles, which is crucial for effective antiviral and immunomodulatory interventions [14]. This study aims to leverage ADMET profiling, network pharmacology, and molecular docking to evaluate the repurposing potential of PN for COVID-19 treatment.

## 2. Materials and Methods

### 2.1. Compound Screening and Preparation

#### ADMET Analysis

Using the SwissADME web server (http://www.swissadme.ch), a validated computational tool for predicting absorption, distribution, metabolism, excretion, and drug-likeness parameters of small molecules, the pharmacokinetic and drug-likeness characteristics of the 27 phytochemicals found in Product Nkabinde (PN) were assessed. The canonical SMILES structures of all phytochemicals were obtained from the PubChem database and submitted separately to SwissADME for evaluation. To evaluate molecular flexibility and permeability, important physicochemical properties were calculated, including molecular weight (MW), topological polar surface area (TPSA), lipophilicity (XlogP3), hydrogen bond donors (HBD), hydrogen bond acceptors (HBA), and the number of rotatable bonds. Using the BOILED-Egg model and support vector machine-based methods integrated inside SwissADME, pharmacokinetic parameters including gastrointestinal (GI) absorption, blood–brain barrier (BBB) permeability, and P-glycoprotein (P-gp) substrate status were predicted. Bioavailability scores were computed to indicate the likelihood of systemic exposure after oral administration, and drug-likeness was assessed using Lipinski’s rule of five to quantify oral bioavailability potential. These computer models provide accurate early-stage screening of phytochemicals for drug development and repurposing applications by predicting pharmacokinetic behavior using fragment-based techniques and experimentally validated quantitative structure–activity relationships (QSARs).

### 2.2. Determination of Common Genes

SMILES representations of the 27 phytochemicals from PN were obtained from PubChem. The Swiss Target Prediction database was utilized to computationally ascertain the target proteins of these inhibitors [15,16]. A total of 1034 genes were extracted and stored in an Excel spreadsheet. The gene dataset was cleaned up, and 390 genes were selected for subsequent analysis. GeneCards, a database that includes information on all gene sets relevant to disorders, was used to predict and identify human proteins linked to COVID-19 [17]. A total of 14,478 proteins (genes) were collected, cleaned, and then stored in an Excel spreadsheet for additional screening on the VENNY website. A Venn diagram showing the proteins that intersect between PN phytochemicals and COVID-19 was generated using VENNY 2.1.0 to identify common proteins [18].

### 2.3. Protein–Protein Interaction (PPI) Network and Hub Genes Analysis

After examining common proteins, the protein–protein interaction (PPI) network was constructed using the Search Tool for the Retrieval of Interacting Genes (STRING) database. For further investigation, the PPI network shows interactions with high confidence [19]. The common genes were uploaded, and the PPI network was computed after selecting Homo sapiens from the drop-down menu and selecting the multiple proteins icon in the STRING database. The examined PPI network was exported and stored in high-resolution Portable Network Graphics (PNG) format. To identify hub genes, the PPI network was also uploaded to Cytoscape (version 3.10.3) [20]. The hub genes, which are involved in key biological processes and maintain the integrity of the PPI network, are often considered important genes. They disclose the mechanistic dynamics of illnesses by altering cellular function. The latest version of Cytoscape, 3.10.3, was downloaded. Cytohubba and yfile were then included in the program. The PPI network was exported from STRING and opened in Cytohubba within Cytoscape to identify hub genes. The MCC and degree-based topological analysis approaches were used to identify hub genes and rank them by centrality and importance in the PPI network.

The ShinyGO 0.85 database was used for Kyoto Encyclopedia of Genes and Genomes (KEGG) and Gene Ontology (GO) pathway analysis [21]. To understand their biological roles, identify potential treatment targets, and elucidate the mechanisms underlying COVID-19, the hub genes were added to the database and their functional enrichment assessed [22,23]. The Benjamini–Hochberg method and the hypergeometric test are used to calculate P-values and False Discovery Rates (FDR) [24]. Fold enrichment is calculated by dividing the percentage of pathway genes among the ten hub genes by the equivalent percentage in the background. The effect magnitude is directly measured as fold enrichment, and statistical significance is indicated by the FDR [25]. This study employed an FDR cut-off of 0.05 to show the 10 most enriched pathways. The average sorting of the pathways is done using FDR and fold enrichment when ‘Select by FDR and Sort by Enrichment’ was selected.

### 2.4. Systems Preparation, Molecular Docking

To predict and assess binding energies for the SARS-CoV-2 Mpro-PN, PLpro-PN, and PN-COVID-19 hub protein omplexes, molecular docking was performed using PyRx. The complexes’ putative multi-targeted molecular activities, which consistently match the network pharmacology structure, are revealed by molecular docking [26]. Moreover, many therapeutic target networks, rather than single-target networks, are revealed by molecular docking. Molecular docking was performed using AutoDock Vina implemented within the PyRx virtual screening platform. The PubChem database was used to obtain the three-dimensional structures of the PN phytochemicals. These structures were then loaded into PyRx, where ligand geometry optimization and energy minimization were carried out using Open Babel with the universal force field (UFF). Prior to docking and optimization, water molecules and co-crystallized ligands were eliminated from the 10 target hub protein structures and the 2 SARS-CoV-2 structures (Mpro and PLpro) that were acquired from the Protein Data Bank (PDB) (PTPN11 PDB ID 6BN5, SRC PDB ID 2SRC, STAT1 PDB ID 1YVL, STAT3 PDB ID 6NUQ, SYK PDB ID 4XG4, HSP90AA1 PDB ID 4R3M, PIK3CB PDB ID 4PUZ, PIK3R1 PDB ID 5XGI, EGFR PDB ID 4R3P, HCK PDB ID 5H0B, Mpro PDB ID 8DZ2, and PLpro PDB ID 7CJM). Molecular docking was performed using AutoDock Vina (v1.1.2) within PyRx 0.8, following a standardized, reproducible protocol. Protein structures were prepared by removing co-crystallized ligands and water molecules, followed by the addition of polar hydrogens and Gasteiger charges, with receptors treated as rigid. Ligands were energy-minimized using Open Babel (UFF) and docked with full torsional flexibility. Docking grids were centered on the crystallographic binding sites and fully covered the active or regulatory pockets, with grid box dimensions shown in Table 1. Docking was performed with an exhaustiveness value of 8, yielding 9 poses per ligand. The pose with the lowest predicted binding free energy and correct pocket occupation was selected for analysis. Redocking validation was performed using the Schrödinger Suite (Maestro v2022-1) to assess the reliability of the docking protocol. For each hub protein, the co-crystallized ligand was extracted from the experimental structure, and the receptor was prepared using the Protein Preparation Wizard with standard settings, including bond-order assignment, hydrogen addition, hydrogen-bond network optimization, and restrained energy minimization. The extracted native ligand was then re-docked into the original binding site using Glide under the same docking parameters applied in the main docking experiments. The resulting docked pose was superimposed onto the crystallographic ligand conformation, and root-mean-square deviation (RMSD) values were calculated over heavy atoms to evaluate pose recovery. The reproduced poses showed acceptable agreement with the experimental conformations, supporting the robustness and reproducibility of the docking workflow. The Vina Wizard was used to construct docking grids that included the active or regulatory binding domains of each target protein. The binding affinities were reported as binding free energy values (kcal/mol) after molecular docking with AutoDock Vina using default exhaustiveness parameters [26,27].

## 3. Results and Discussion

### 3.1. ADMET Profiling

To evaluate molecular flexibility and permeability, important physicochemical properties were calculated, including molecular weight (MW), topological polar surface area (TPSA), lipophilicity (XlogP3), hydrogen bond donors (HBD), hydrogen bond acceptors (HBA), and the number of rotatable bonds. Other pharmacokinetic parameters, including gastrointestinal (GI) absorption, blood–brain barrier (BBB) permeability, and P-glycoprotein (P-gp) substrate status, were predicted. The in silico ADMET assessment of PN phytochemicals reveals advantageous pharmacokinetic properties, indicating potential for repurposing as COVID-19 interventions. Many of the PN bioactive compounds, such as quercetin, catechin, epicatechin, emodin, chrysophanol, physcion, gallic acid, and diosgenin, demonstrated elevated anticipated gastrointestinal absorption and adherence to Lipinski’s rule of five, signifying favorable oral bioavailability and drug-like properties as illustrated in Table 2. Compounds like 7,7′-dihydroxy-3,8′-biscoumarin, prostratin, quercetin, and catechin exhibited no Lipinski violations and favorable lipophilicity (XlogP3 < 5), indicating optimal membrane permeability and systemic distribution.

Moreover, bioavailability scores of 0.55 for numerous PN ingredients indicate a high likelihood of achieving therapeutically relevant plasma concentrations. These pharmacokinetic characteristics align with contemporary repurposing frameworks that prioritize oral bioavailability, a molecular weight under 500–600 g/mol, and a balanced polarity for antiviral drug candidates [12,28]. High gastrointestinal absorption and low lipophilicity enable rapid systemic exposure, which is essential for blocking early SARS-CoV-2 replication and reducing disease progression [29]. Moreover, the ADMET profile demonstrates pharmacologically beneficial transport and distribution properties, further encouraging the repurposing of PN. Predicted interactions of selected phytochemicals, including epicatechin, catechin, and prostratin, with P-gp transporters imply regulated intracellular accumulation and advantageous tissue distribution. In contrast, compounds like diosgenin and chrysophanol exhibited anticipated permeability across the blood–brain barrier, suggesting potential significance in alleviating SARS-CoV-2-related neurological symptoms. These findings are relevant given accumulating evidence that COVID-19 pathophysiology involves multi-organ and neuroinflammatory mechanisms driven by viral invasion and cytokine dysregulation [30,31]. Furthermore, the balanced physicochemical properties and moderate to high bioavailability scores of PN phytochemicals complement existing drug repurposing approaches targeting host–pathogen interaction pathways, such as viral entry inhibition, oxidative stress regulation, and inflammatory signaling. When taken as a whole, these ADMET features support PN’s translational potential as an orally bioavailable, pharmacokinetically viable candidate for repurposing in COVID-19 therapy and support additional in vitro, in vivo, and clinical validation [14,32].

### 3.2. Intersection Analysis of COVID-19 and PN Target Proteins

The Venn diagram in Supplementary Figure S1 illustrates the overlap between the predicted protein targets of bioactive phytochemicals in PN (110) and the host genes associated with COVID-19 (14,207). It highlights 271 overlapping genes (1.9%) that represent a shared molecular space, which may be actionable in the context of SARS-CoV-2 infection. Given that modulators of multiple pathways can exert disproportionate influence on disease phenotypes beyond what simple target counts would suggest, systems pharmacology and network medicine frameworks acknowledge that even slight overlaps between a disease gene set and a compound’s target profile may be biologically significant when the intersecting nodes occupy central regulatory or hub positions within protein–protein interaction networks. In particular, network pharmacology suggests that therapeutics acting at multiple nodes (polypharmacology) can more effectively restore network homeostasis and that complex diseases, such as COVID-19, involve dysregulation across interconnected biological pathways, including cytokine signaling, innate immune responses, and host kinase cascades, rather than singular aberrations [33,34]. Hundreds of host factors that SARS-CoV-2 uses for entry, replication, and immune evasion have been identified in the COVID-19 disease interaction network. Immune signal transducers, kinases, and transcriptional regulators play crucial roles in this process, and their disruption is linked to the severity of the illness and unfavorable outcomes [35]. Therefore, if enriched for such network hubs and signaling mechanisms, the 271 overlapping targets in the Venn diagram can form a mechanistically rich subset, even if they only comprise a small percentage of the entire COVID-19 gene set. The idea that PN’s phytochemicals may interact with host pathways important to COVID-19 pathogenesis is supported by this conceptual basis, which also validates the network pharmacology technique employed in this study and suggests the need for additional mechanistic research.

### 3.3. Protein-Protein Interaction (PPI) Network of Shared COVID-19-PN Targets

The PPI network shown in Supplementary Figure S2 illustrates a dense interactome of the 271 shared targets between COVID-19-related pathways and PN-predicted targets, highlighting that these targets are not isolated elements but components of an integrated biological system involving host cellular functions. A densely connected core of proteins is revealed by the protein–protein interaction (PPI) network derived from the intersection of COVID-19–related host genes and anticipated targets of PN phytochemicals, highlighting the intricate molecular interactions underlying SARS-CoV-2 pathogenesis and the host response. Hub proteins and signaling modules that coordinate important biological processes, including cytokine signaling, innate immune activation, and cell stress responses, are frequently highlighted by such tightly connected networks. PPI network hubs are attractive targets for multi-target therapies and drug repurposing initiatives because recent systems biology research has shown that they frequently represent functional bottlenecks in disease etiology, where perturbations can spread changes across multiple pathways [1,36].

### 3.4. Identifying COVID-19-PN Hub Proteins

**Figure 1:**
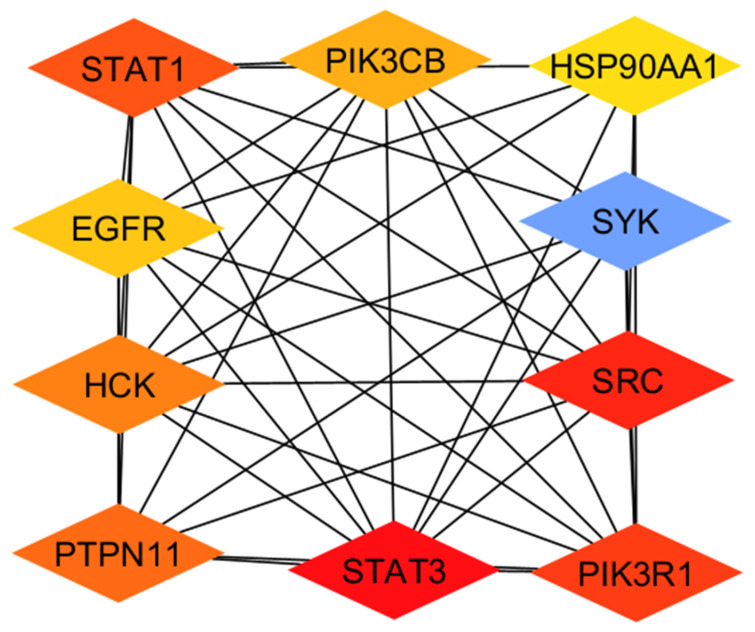
Hub gene interaction network highlighting key COVID-19 host targets (STAT1, STAT3, SRC, HCK, SYK, EGFR, PIK3CB, PIK3R1, HSP90AA1, and PTPN11) implicated in immune signaling and inflammatory regulation and prioritized for PN repurposing analysis.

Figure 1 illustrates the core hub gene network derived from the protein–protein interaction analysis, highlighting STAT1, STAT3, SRC, HCK, SYK, EGFR, PIK3CB, PIK3R1, HSP90AA1, and PTPN11 as central regulatory nodes in COVID-19 pathogenesis.

The severity and progression of SARS-CoV-2 infection are determined by these genes, which are well-known mediators of immunological signaling, inflammatory control, kinase activity, and viral-host interactions. While SRC, HCK, and SYK regulate immune cell activation and inflammatory amplification, STAT1 and STAT3 are crucial components of interferon and cytokine signaling pathways. HSP90AA1 stabilizes several target proteins implicated in stress and immunological responses, while EGFR and PI3K pathway members (PIK3CB and PIK3R1) regulate cellular survival, metabolism, and viral replication. Phosphatase-dependent signaling balance in these networks is further regulated by PTPN11. These hub genes are classified as high-value therapeutic targets in COVID-19 due to their dense interconnection, indicating their functional dependency. Crucially, rather than relying solely on single-target antiviral therapy, the discovery of these hubs provides a solid molecular basis for repurposing PN as a multi-target therapeutic capable of modulating key host signaling cascades [35,36,37]. Functional and clinical implications of the identified hub genes are illustrated in Table 3 below.

### 3.5. Molecular Docking Analysis

In this study, molecular docking of the hub genes and 27 PN phytochemicals was performed in PYRX 0.8. All co-crystallized ligands were redocked into their corresponding protein targets using the same AutoDock Vina (version 1.1.2) parameters to ensure accurate docking and enable benchmarking of PN phytochemicals. Native ligands were redocked using the same grid box coordinates and exhaustiveness settings after being extracted from crystallographic complexes. Table 1 and Table 4 illustrate AutoDock Vina grid box parameters used for redocking and molecular docking results of PN-COVID-19 Hub Genes.

The potential of PN phytochemicals for repurposing as a host-directed treatment for SARS-CoV-2 infection is supported by the favorable multi-target interactions observed in the molecular docking investigation of PN phytochemicals against targeted COVID-19 hub genes illustrated in Table 4. In comparison to co-crystallized ligands, several PN compounds exhibited greater or similar binding affinities across key host targets involved in viral pathogenesis and immunological signaling. For example, PIK3R1 demonstrated a much higher binding affinity with Pimelea factor P2 (−11.0 kcal/mol vs. −8.2 kcal/mol), indicating successful interaction with PI3K signaling, survival, and inflammatory dysregulation pathways that are dysregulated in COVID-19 immunological responses [74]. The significance of HSP90 as a molecular chaperone that stabilizes viral and host stress proteins and promotes SARS-CoV-2 pathogenicity is supported by the increased binding of 7,7′-dihydroxy-3,8′-biscoumarin to HSP90AA1 [75]. The strong affinities of procyanidin B2 toward HCK (−10.5 kcal/mol) and rutin toward SRC (−10.5 kcal/mol) further suggest that PN may control SRC-family kinases, which are essential for inflammatory amplification and macrophage activation during severe viral pneumonia. Similarly, enhanced gnidicin docking to SYK is consistent with data that SYK inhibition significantly reduces pulmonary damage in SARS-CoV-2 infection and that SYK signaling enhances immune-complex-mediated lung inflammation. Stronger binding of diosgenin to PIK3CB and EGFR suggests that immunometabolic and epithelial signaling pathways linked to lung damage, viral replication, and post-COVID fibrosis may be regulated [76,77,78]. SARS-CoV-2 is known to suppress STAT1 activation and skew signaling toward STAT3-driven inflammation, thereby impairing antiviral interferon responses and promoting a cytokine storm. Although PN phytochemicals demonstrated slightly weaker affinities toward STAT1 and STAT3, their stable interactions remain biologically significant. When considered as a whole, these docking results reveal a polypharmacological mechanism in which PN phytochemicals interact with multiple linked host targets, rather than a single node. This strategy is gaining increasing recognition as beneficial for complex diseases, such as COVID-19, where host immune dysregulation and viral replication coexist. The repurposing of PN as a multi-target therapeutic option for COVID-19 treatment is well supported by our computational and molecular findings [34,36,38,39,79].

To fully evaluate the antiviral potential of PN, additional docking studies were conducted on the viral main protease (Mpro) and papain-like protease (PLpro) after the phytochemicals were molecularly docked to priority COVID-19 host hub genes. Effective treatment interventions also require direct inhibition of the viral replication machinery, even though host-target docking provides crucial insight into the capacity of PN agents to influence dysregulated immunological, inflammatory, and signaling pathways associated with COVID-19 pathogenesis. While PLpro promotes viral maturation and concurrently inhibits host antiviral responses by deubiquitination and interferon antagonism, SARS-CoV-2 depends on Mpro to cleave viral polyproteins into functional non-structural proteins required for replication complex assembly. It has been demonstrated that inhibiting these proteases substantially suppresses viral replication and enhances antiviral results in experimental systems. They are regarded as critical and highly conserved viral targets [35,80]. Therefore, a thorough systems-level evaluation of the therapeutic potential of PN phytochemicals is provided by testing them against both host hub proteins and viral proteases. This supports a dual host-directed and virus-directed repurposing strategy for the treatment of COVID-19.

### 3.6. Overview of Docking Affinities Against Viral Proteases and Host Hub Targets

The binding affinities of PN phytochemicals to the critical SARS-CoV-2 viral proteases, main protease (Mpro) and papain-like protease (PLpro), are also investigated. The molecular docking results are illustrated in Table 5 and Table 6 below.

The molecular docking investigation in Table 5 demonstrates that numerous phytochemicals from PN exhibit significant binding affinities for the SARS-CoV-2 main protease (Mpro) compared to the co-crystallized inhibitor. Oleanolic acid showed the highest binding affinity (−12.9 kcal/mol), significantly exceeding the reference ligand (−8.3 kcal/mol). Epigallocatechin gallate also showed markedly enhanced binding (−9.4 kcal/mol), while quercetin-3-O-β-D-(6′-galloyl)-glucopyranoside (−8.6 kcal/mol) exhibited comparable or slightly improved affinities relative to the control, indicating stable and favorable interactions within the Mpro active site. Altogether, these results demonstrate PN’s multi-compound inhibitory activity, with several phytochemicals exhibiting binding profiles comparable to or superior to those of the native ligand. This supports the hypothesis that PN could function as a promising natural source of SARS-CoV-2 Mpro inhibitors and calls for further in silico and experimental validation.

The docking analysis against SARS-CoV-2 papain-like protease (PLpro) in Table 6 below shows that all evaluated phytochemicals had higher binding affinities than the co-crystallized reference ligand, which is consistent with the aim of repurposing PN for COVID-19 treatment through an integrated network pharmacology and molecular docking framework. The highest binding affinity (−8.4 kcal/mol) was exhibited by quercetin-3-O-β-D-(6′-galloyl)-glucopyranoside, which was closely followed by procyanidin B2 and 2,4′,6-trihydroxy-4-methoxybenzophenone-2-O-glucoside, indicating a better projected inhibitory potential toward PLpro. These potent binding interactions suggest that PN phytochemicals may effectively inhibit both viral replication and host immunological regulation, as PLpro is crucial for viral polyprotein cleavage and immune evasion [81]. Furthermore, the multi-target, multi-component therapeutic hypothesis underlying PN is supported by the consistency between docking validation against PLpro and network-derived hub target predictions. The formulation’s synergistic antiviral potential is highlighted by the superior binding profiles of polyphenolic chemicals, triterpenoids, and flavonoid glycosides. These results collectively suggest that PN has potential as an anti-COVID-19 treatment and provide a solid computational basis for further in vitro, in vivo, and clinical studies.

Furthermore, to assess ranking robustness, independent redocking validations were performed in Schrödinger Maestro 2022-1, in which top-ranked phytochemicals consistently retained their relative rankings; detailed results are provided in Supplementary Table S1. In Schrodinger 2022-1, the root-mean-square deviation (RMSD) between redocked poses and their experimental crystal conformations, after protein backbone superposition, was used to assess docking pose accuracy. The visual superpositions of crystallographic and redocked ligand poses in Supplementary Figures S3–S14 allow direct visual assessment of pose recovery in addition to numerical RMSD values. This study’s molecular docking methodology was not intended to predict absolute binding affinity, but rather to offer comparative structural insights into the multitarget interaction landscape of PN phytochemicals. As a result, protocol validation focused on experimentally grounded structural accuracy, using RMSD-based pose-recovery analysis (Table 7) and explicit redocking of co-crystallized ligands, which verified consistent replication of known binding modes across hub targets and SARS-CoV-2 proteases. Structural reliability of the docking workflow was ensured through explicit redocking and RMSD-based validation against crystallographic ligand conformations. Given the multitarget and mechanistic focus of this network pharmacology study, this combined structural validation approach was considered appropriate for prioritizing ligand–target interactions and guiding pathway-level interpretation. To ensure transparent evaluation of docking performance and ligand selectivity, binding affinity values for all PN-hub genes are provided in Supplementary Table S2. These score distributions reveal consistent ranking patterns across ligands and targets, supporting the robustness of the highlighted top interactions. These results indicate that, despite structural diversity, PN phytochemicals occupy a relevant physicochemical space for protein interactions. Accordingly, docking outcomes are interpreted as indicative of relative binding propensity rather than definitive structural binding modes.

### 3.7. Functional Enrichment Analysis

**Figure 2:**
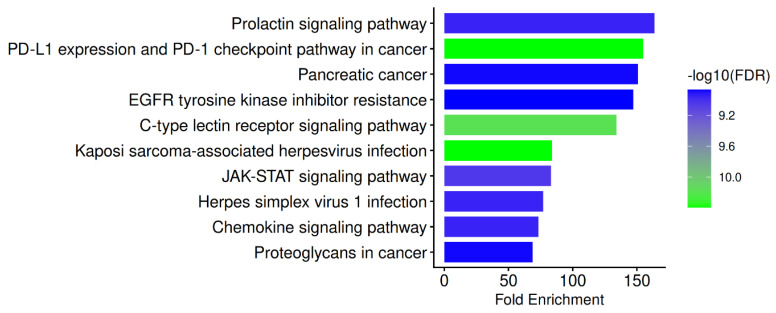
KEGG Pathway Enrichment of PN–COVID-19 Hub Genes.

All the gene sets in the Pathway database menu are selected and investigated separately. These include Gene Ontology (GO) biological process, GO cellular component, GO molecular function, and the Kyoto Encyclopaedia of Genes and Genomes (KEGG). Using the FDR cut-off (0.05), the minimum pathway size (2), and the maximum pathway size (2000), the most important COVID-19 routes were found. The results are illustrated in Table 8 and Figure 2 and discussed below.

COVID-19 is increasingly recognized as a multi-layered disease in which SARS-CoV-2 replication, innate immune sensing, cytokine amplification, immune exhaustion, endothelial damage, and dysregulated tissue repair programs interact to determine clinical severity and post-acute complications [82]. The scientific case for repurposing candidates with coordinated, multi-target activity across viral and host nodes is strengthened by recent therapeutic reviews that highlight how viral evolution and heterogeneous host responses can undermine reliance on single-pathway or single-target strategies [83]. The host-directed immunomodulatory concept for PN is mechanistically consistent with the enrichment of immune and signaling pathways associated with the ten hub genes (STAT1, STAT3, SRC, HCK, EGFR, SYK, PIK3CB, PIK3R1, HSP90AA1, and PTPN11), in addition to direct antiviral targeting of SARS-CoV-2 proteases. One of the main basic characteristics of severe COVID-19 is dysregulated cytokine signaling, and systemic complications, including acute respiratory distress syndrome (ARDS), are caused by hyperinflammation. The central signaling backbones of cytokine-driven biology in severe disease are still highlighted by recent mechanistic syntheses [84]. Recent COVID-19-focused reviews and systematic analyses describe how aberrant JAK/STAT activation contributes to immune hyperactivation and cytokine storm phenotypes. The JAK–STAT axis is frequently implicated as a crucial intracellular pathway through which interferons and inflammatory cytokines shape antiviral defence and inflammatory progression [85]. Specifically, disease models often differentiate between disproportionate STAT3-associated inflammatory effects and suppressed STAT1-linked antiviral interferon interventions. This pattern is consistent with clinical observations of impaired antiviral control along with the adverse effects of inflammation in severe cases [86]. Strong docking interactions between PN phytochemicals and STAT1/STAT3 in this context provide support for the hypothesis that PN may alter this equilibrium, thereby strengthening antiviral signaling while reducing pathological cytokine amplification, an effect traditionally sought by host-directed interventions in COVID-19 [87].

Another factor repeatedly shown to influence lung immunopathology is chemokine-driven leukocyte influx. Elevated chemokines, including C–C Motif Chemokine Ligand 2 (CCL2) and C–X–C Motif Chemokine Ligand 10 (CXCL10), have been observed in COVID-19, and recent reviews and integrative analyses have linked their dysregulation to disease severity, immune-cell trafficking patterns, and the inflammatory burden [88]. The idea that increased chemokine signaling may stimulate excessive influx into inflammatory tissues and exacerbate pulmonary injury is supported by another recent study on monocyte–macrophage dynamics [89]. Considering that these kinases link receptor interaction to immune-cell activation, migration, and effector programs, the presence of SRC-family and related kinases (SRC, HCK, SYK) among the hub genes provides mechanistic coherence. Therefore, the docking-supported engagement of PN phytochemicals with these kinase hubs is consistent with a physiologically based approach to minimize detrimental inflammatory cell influx and activation while maintaining essential antiviral responses [90]. Current understanding of early myeloid sensing and inflammatory programming during viral infection is also consistent with the enrichment of innate immune recognition pathways centered on C-type lectin receptors (CLRs). CLRs are essential pattern-recognition receptors on myeloid cells that recognize a broad range of ligands and exert potent downstream inflammatory and immunoregulatory effects, according to a recent immunology review [91]. Furthermore, SYK is a canonical signaling mediator downstream of several CLR families, making it a convergence point where modification may alter the strength of myeloid activation and cytokine production. By reducing maladaptive myeloid activation without the need for direct interference with a single upstream receptor, the combination of CLR-linked pathway relevance and PN’s anticipated strong binding to SYK provides a mechanistic bridge from pathway enrichment to a host-directed immunomodulatory rationale [87,92].

Severe acute illness and chronic post-acute symptoms are increasingly associated with adaptive immune dysfunction and exhaustion, often characterized by the overexpression of inhibitory receptors. Exhausted T-cell phenotypes, which are defined by reduced effector function and prolonged inhibitory receptor expression, are specifically connected to severe COVID-19, delayed viral clearance, and persisting symptoms consistent with long-term COVID, according to a recent review [93]. Checkpoint-like immune attenuation may persist beyond the acute phase, as evidenced by human studies showing sustained expression of inhibitory markers, such as PD-1, in individuals with prolonged COVID-19 symptoms months after infection [94]. Given that STAT3 and PI3K-linked signaling play major roles in determining T-cell differentiation stages, survival, and functional exhaustion trajectories, these findings reinforce the significance of PD–1/PD–L1–related pathway enrichment in the context of repurposing PN. Consequently, multi-node modulation of upstream signaling hubs (STATs, SRC-family kinases, and components of the PI3K complex) provides a logical mechanistic pathway through which PN could support the restoration of effective antiviral immunity while reducing immune exhaustion patterns associated with chronic illness [93,94].

As host pathways co-opted during SARS-CoV-2 infection to enhance cellular states favorable to viral proliferation or stress tolerance, growth factor receptor and survival signaling networks, particularly EGFR and PI3K/AKT, have also garnered interest. Clinical-translational research has investigated the inhibitory potential of EGFR pathway targeting against SARS-CoV-2 variants, and experimental and mechanistic data indicate that SARS-CoV-2 can activate EGFR-mediated survival signaling early in the infection process. Furthermore, due to its roles in host survival signaling, inflammation, and virus–host contact networks, evaluations of the PI3K/Akt/mTOR axis have characterized it as a potential pharmaceutical target in COVID-19 [95,96]. The suggestion that PN may function as a host-directed modulator of kinase networks linked to severe inflammatory injury and maladaptive repair responses is thus supported by the hub inclusion of EGFR along with PIK3R1 and PIK3CB, which offers a mechanistically consistent map from pathway enrichment to target-level docking signals [97].

The biology of the extracellular matrix and cell-surface glycans, as reflected in proteoglycan-related pathway signals, is directly linked to the entry and tropism of SARS-CoV-2. Independent, high-impact experimental evidence suggests that the SARS-CoV-2 spike protein binds heparan sulfate in a sequence-dependent manner, corroborating a model in which heparan sulfate proteoglycans serve as initial attachment factors that enhance viral engagement and subsequent receptor interactions [98]. Another recent study further substantiates the functional roles of heparan sulfate proteoglycans in modulating spike conformation and facilitating infection-related interactions within endothelial environments, thereby emphasizing the translational significance of proteoglycan biology to the vascular and inflammatory aspects of COVID-19 [99]. These results support the interpretation of proteoglycan-associated enrichment in the literature as biologically significant, suggesting that viral attachment efficiency and downstream inflammatory signaling landscapes may be influenced by host pathways that involve glycan–receptor–signaling interfaces [100].

The enrichment of prolactin signaling can also be understood in terms of its recognized immunoregulatory functions. Contemporary reviews describe prolactin as a multifaceted immunomodulatory hormone that regulates cytokine production and immune cell activity. Studies on COVID-19 further demonstrate its association with inflammatory responses that correlate with disease severity [101,102]. Consequently, prolactin-driven effects intersect with STAT-, SRC-, and PI3K-linked signaling biology; the co-occurrence of prolactin-related pathway enrichment with hubs such as STAT1/STAT3, SRC/HCK, and PI3K complex components supports the mechanistic potential that PN’s multi-target profile could influence endocrine–immune interactions that modulate inflammatory activity in COVID-19 [101].

The pathway enrichment profile, which is backed by recent virology, immunology, and clinical-translational research, is consistent with a coherent repurposing narrative that includes direct antiviral pressure on critical SARS-CoV-2 proteases (Mpro and PLpro) and host-directed modulation of immune signaling circuits controlling innate sensing (CLR–SYK), cytokine amplification (JAK–STAT), inflammatory trafficking (chemokines), immune exhaustion (PD-1–associated T-cell dysfunction), and kinase-driven survival/injury programs (EGFR–PI3K) [81]. In line with current host-directed antiviral development strategies, this trend suggests prioritizing PN for experimental validation using enzymatic inhibition assays for Mpro/PLpro, cell-based infection models with cytokine/chemokine profiling, and mechanistic immunophenotyping focused on the STAT1/STAT3 balance, SYK-mediated myeloid activation states, and dynamics of exhaustion markers [90].

### 3.8. Gene Ontology (GO) Biological Process, GO Cellular Component, GO Molecular Function

**Figure 3:**
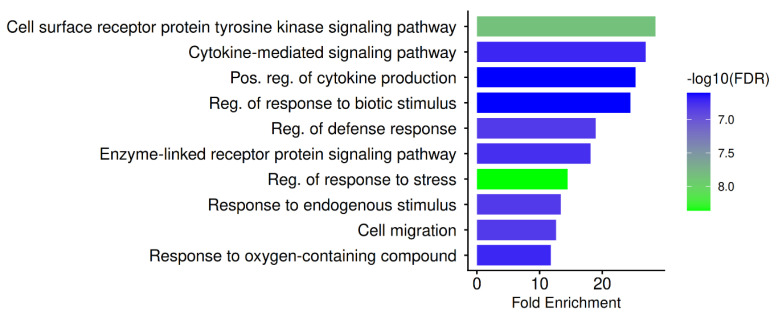
GO biological process for PN-COVID-19 hub genes.

PN-associated targets focus on biological processes crucial to SARS-CoV-2 pathogenesis and host disease development, as indicated by the GO biological process enrichment profile illustrated in Figure 3. The predominant importance of receptor-driven kinase cascades in COVID-19 inflammation, immunological activation, and tissue damage is reflected in the greatest enrichment in cell surface receptor protein tyrosine kinase signaling and enzyme-linked receptor protein signaling. Cytokine receptors and receptor tyrosine kinases regulate PI3K, JAK–STAT, SRC, and MAPK signaling, all of which are frequently associated with the biology of cytokine storms and the severity of COVID-19 [103,104]. The concept that PN operates on upstream regulatory nodes capable of simultaneously modifying several downstream inflammatory and antiviral programs is supported by the enrichment of these activities.

The recent perspective of COVID-19 as a cytokine-driven immunopathology is further supported by robust evidence of cytokine-mediated signaling, positive modulation of cytokine production, and regulation of the defensive response. Respiratory failure and systemic consequences are closely associated with elevated IL-6, TNF-α, IFN-γ, and chemokines, which are indicative of severe disease [105,106,107]. A host-directed immunomodulatory approach that may reduce hyperinflammation while maintaining antiviral competence is supported by the GO biological process enrichment, which indicates that PN targets engage in regulatory layers that control cytokine amplitude rather than isolated cytokine molecules. Immune-cell trafficking into lung tissue, a key factor in COVID-19 pathogenesis, is mechanistically consistent with the enrichment of cell migration. Lung damage and ARDS are directly linked to the excessive recruitment of neutrophils, macrophages, and monocytes [44,108]. Therefore, PN may be able to reduce tissue-destructive immune infiltration while preserving host defence by focusing on migratory regulators.

The biological processes triggered by viral infection, oxidative stress, and inflammatory injury are reflected in the enrichment of responses to biotic stimuli, defense responses, and stress responses. According to Cavalcanti et al. (2022) [109], SARS-CoV-2 infection increases endothelial dysfunction, immunological activation, and tissue damage by inducing oxidative stress and stress-response signaling. These GO terms suggest that PN may influence cellular stress adaptation processes, which are essential for preventing the progression of viral infection to organ failure [109].

**Figure 4:**
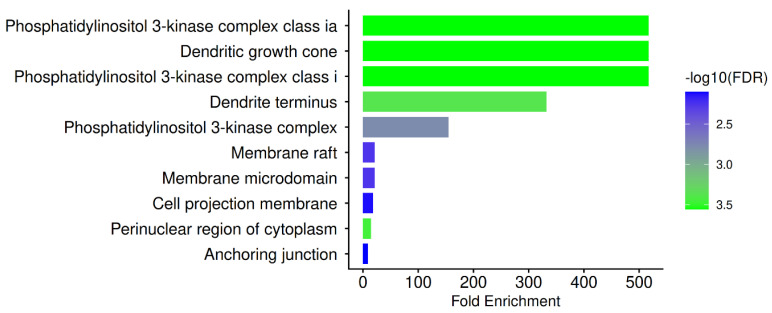
GO cellular component for PN-COVID-19 hub genes.

A key intracellular signaling hub is highlighted at the GO cellular component level (Figure 4) by the predominant enrichment of the phosphatidylinositol 3-kinase (PI3K) complex, specifically the class I/IA subfamily. The PI3K complex serves as a master regulator of cytokine signaling, metabolism, support mechanisms for viral replication, and immune cell survival. Modulation of the PI3K/AKT signaling pathway has been proposed as a potential therapeutic approach for COVID-19 and is frequently associated with SARS-CoV-2 infection and inflammation [110,111]. The significance of PN’s hub targets PIK3R1 and PIK3CB as molecular anchors for host-directed treatment is clearly reinforced by the enrichment of this complex.

Furthermore, localization to membrane microdomains and membrane rafts has biological significance. Signal transmission, receptor clustering, and viral entry all depend on lipid rafts. Raft organization is essential for both immune receptor signaling and SARS-CoV-2 entry [112]. The idea that PN targets participate in spatially organized signaling platforms that control immune receptor signaling, cytokine amplification, and viral attachment is supported by their enrichment in these compartments. Perinuclear cytoplasm enrichment is compatible with intracellular trafficking of transcriptional regulators, host signaling complexes, and viral components. In perinuclear areas where innate immune signaling and stress responses are coordinated, the replication of SARS-CoV-2 and the signaling of host responses converge [113]. Therefore, possible modulation of viral–host signaling integration is supported by PN’s anticipated activity in this cellular compartment.

PN targets are further linked to endothelial and epithelial barrier control through the enrichment of anchoring junctions and cell projection membranes. COVID-19 is increasingly recognized as a vascular and barrier disease, with junctional disruption leading to edema, thrombosis, and inflammation [105,106]. The possible protective function of PN against barrier malfunction is supported by the regulation of these structures.

**Figure 5:**
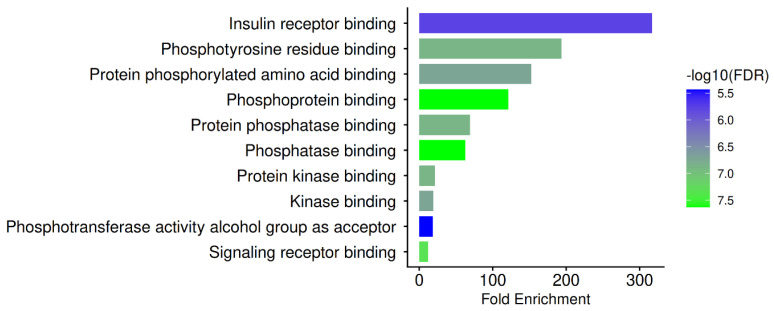
GO molecular function for PN-COVID-19 hub genes.

A signaling-centric pharmacological profile is revealed at the GO molecular function level (Figure 5) by the predominant enrichment in phosphotyrosine residue binding, phosphoprotein binding, kinase binding, and phosphatase binding. Numerous phosphorylation-dependent signaling cascades regulate cytokine release, immune activation, and cell survival, thereby driving COVID-19 pathogenesis [114,115]. The capacity of PN targets to bind phosphorylated proteins and kinases suggests that PN may not be a single-receptor inhibitor but rather a modulator of the signaling network. Considering that phosphatases control the termination and fine-tuning of inflammatory signals, the enrichment of protein phosphatase-binding sites and phosphatase-binding sites is highly significant. In viral infections, dysregulated phosphatase activity has been associated with immunological imbalance and chronic inflammation [116]. Thus, a mechanism for re-establishing signaling equilibrium is suggested by PN’s interaction with phosphatase-associated nodes.

Given that insulin resistance and metabolic dysfunction significantly impact disease severity and inflammatory responses, the enrichment of insulin receptor binding further suggests its relevance to COVID-19 [117]. The biological coherence of PN’s GO enrichment network is strengthened by the intersections of insulin receptor signaling with PI3K/AKT pathways. Moreover, a broad ability to modulate receptor-driven immunological and inflammatory processes is reflected in enrichment for signaling receptor binding. This is in line with a multi-target immunomodulatory profile that can coordinate responses across immunological checkpoints, growth factor receptors, and cytokine receptors, all of which are essential for the progression of COVID-19 disease [118].

Repurposing PN as a host-directed and antiviral treatment candidate for COVID-19 is strongly supported by the GO Biological Process, Cellular Component, and Molecular Function enrichment patterns taken together. PN specifically targets the molecular systems known to control viral replication efficiency, inflammatory escalation, immune exhaustion, and tissue injury, as evidenced by the convergence on receptor-driven kinase signaling, cytokine regulation, stress adaptation, immune-cell migration, PI3K complex localization, membrane raft signaling, and phosphorylation-dependent molecular interactions. These GO results indicate that PN is a biologically coherent, multi-node treatment candidate, with strong support for experimental and clinical validation in COVID-19 management, as evidenced by molecular docking against SARS-CoV-2 proteases and host signaling hubs.

## 4. Conclusions

This study provides a systems-level evaluation of the potential repurposing of Product Nkabinde (PN) as a multi-target therapeutic candidate for COVID-19. ADMET profiling demonstrated that several PN phytochemicals possess favorable pharmacokinetic and drug-likeness properties, supporting their suitability for further investigation. Network pharmacology analysis revealed that PN compounds interact with key host signaling proteins implicated in COVID-19 pathogenesis, including STAT1, STAT3, SRC, HCK, SYK, EGFR, PIK3CB, PIK3R1, HSP90AA1, and PTPN11. Molecular docking further showed strong binding affinities of several phytochemicals toward both viral proteases (Mpro and PLpro) and host regulatory targets, suggesting a coordinated multi-target mechanism that may influence viral replication, immune signaling, and inflammatory responses. Functional enrichment analyses supported these findings by identifying significant involvement of pathways such as JAK–STAT signaling, chemokine signaling, PD-1/PD-L1 checkpoint regulation, and PI3K signaling, which are closely associated with antiviral immunity and COVID-19 immunopathology. Collectively, these results suggest that PN may exert both direct antiviral and host-directed immunomodulatory effects, highlighting its potential as a multi-component therapeutic candidate. While these findings provide a strong computational basis for repurposing PN, further experimental validation through enzymatic assays, cell-based infection models, and preclinical studies will be necessary to confirm its antiviral efficacy and therapeutic safety.

## Figures and Tables

**Table 1:** AutoDock Vina grid box parameters used for redocking and phytochemical docking.

Target Protein	PDB ID	Grid Center (Å)	Grid Size (Å)	Exhaustiveness	Number of Poses
STAT1	1YVL	X = −9.4601 Y = −46.2330 Z = 193.8382	X = 99.8062 Y = 58.9802 Z = 115.5129	8	9
STAT3	6NUQ	X = −2.2294 Y = 19.1326 Z = 24.6217	X = 72.3191 Y = 115.0291 Z = 91.5312	8	9
HSP90AA1	4R3M	X = −32.9405 Y = −14.8778 Z = −20.5203	X = 42.4899907 Y = 43.8127379 Z = 45.2056279	8	9
PIK3CB	4PUZ	X = −30.1911 Y = −0.2475 Z = 58.7412	X = 45.8255 Y = 55.3426 Z = 60.3925	8	9
PIK3R1	5XGI	X = 17.8179 Y = 34.1451 Z = 30.0453	X = 88.4854 Y = 111.0938 Z = 101.1489	8	9
EGFR	4R3P	X = −57.2821 Y = −7.9282 Z = −24.8951	X = 50.3781 Y = 64.6770 Z = 56.2785	8	9
SRC	2SRC	X = 20.6723 Y = 33.8852 Z = 67.4994	X = 64.5127 Y = 68.7439 Z = 60.9835	8	9
HCK	5H0B	X = 5.7224 Y = −2.5091 Z = −15.9866	X = 59.6835 Y = 81.7633 Z = 73.4306	8	9
SYK	4XG4	X = 12.9037 Y = −11.6072 Z = 17.6213	X = 46.9783 Y = 59.2202 Z = 55.6463	8	9
PTPN11	6BN5	X = 64.0642 Y = 91.0075 Z = 17.1150	X = 60.5899 Y = 74.2658 Z = 60.3082	8	9

**Table 2:** In silico ADMET and drug-likeness profiling of PN phytochemicals.

Phytochemical	MW (g/mol)	TPSA	XlogP3	HBD	HBA	Rot Bonds	Lipinski Violation	GI Abs	BBB	P-gp	Bioavailability
Quercetin-3-O-β-D-(6′-galloyl)-glucopyranoside	616.48	277.27	0.98	10	16	7	3	Low	No	No	0.17
7,7′-Dihydroxy-3,8′-biscoumarin	322.27	100.88	2.81	2	6	1	0	High	No	No	0.55
Prostratin	390.47	104.06	0.7	3	6	3	0	High	No	Yes	0.55
6-(8″-Umbelliferyl)-apigenin	322.27	100.88	2.46	2	6	1	0	High	No	No	0.55
Pimelea factor P2	638.79	127.21	6.15	3	9	5	1	Low	No	Yes	0.55
Wikstroelide A	642.78	144.28	5.33	3	10	14	1	Low	No	Yes	0.55
Gnidicin	628.67	144.28	3.16	3	10	7	1	Low	No	Yes	0.55
Gnidilatidin	648.74	144.28	4.82	3	10	11	1	Low	No	Yes	0.55
Gnidimacrin	774.89	173.74	6.01	4	12	9	2	Low	No	Yes	0.17
(−)-Epicatechin	290.27	110.38	0.36	5	6	1	0	High	No	Yes	0.55
Diosgenin	414.62	38.69	5.67	1	3	0	1	High	Yes	No	0.55
Oleanolic acid	456.7	57.53	7.49	2	3	1	1	Low	No	No	0.85
Procyanidin B2	578.52	220.76	2.37	10	12	3	3	Low	No	No	0.17
Epigallocatechin gallate	458.37	197.37	1.17	8	11	4	2	Low	No	No	0.17
Quercetin	302.24	131.36	1.54	5	7	1	0	High	No	No	0.55
Catechin	290.27	110.38	0.36	5	6	1	0	High	No	Yes	0.55
Emodin	270.24	94.83	2.72	3	5	0	0	High	No	No	0.55
Daucosterol	576.85	99.38	7.74	4	6	9	1	Low	No	No	0.55
β-Sitosterol	414.71	20.23	9.34	1	1	6	1	Low	No	No	0.55
Rutin	610.52	269.43	−0.33	10	16	6	3	Low	No	Yes	0.17
Chrysophanol	254.24	74.6	3.53	2	4	0	0	High	Yes	No	0.55
Physcion	284.26	83.83	3.04	2	5	1	0	High	No	No	0.55
Gallic acid	170.12	97.99	0.7	4	5	1	0	High	No	No	0.56
Quercetin-3-O-arabinoside	434.35	190.28	0.43	7	11	3	2	Low	No	No	0.17
Aloin	418.39	167.91	−0.12	7	9	3	1	Low	No	No	0.55
2,4′,6-Trihydroxy-4-methoxybenzophenone-2-O-glucoside	430.36	141.34	3.92	4	8	2	0	Low	No	No	0.55
2,3,4′,5,6-Pentahydroxybenzophenone-4-C-glucoside	422.38	166.14	0.66	6	10	6	1	Low	No	No	0.55

**Table 3:** Functional roles, COVID-19-specific molecular implications, and clinical relevance of the ten prioritized hub genes identified from the PN-COVID-19 network pharmacology analysis.

Common Name	Full Name	Function	Specific COVID-19 Association	Clinical Relevance	References
STAT1	Signal Transducer and Activator of Transcription 1	Mediator of interferon-driven antiviral gene expression	SARS-CoV-2 inhibits STAT1 activation, impairing antiviral immunity	Biomarker for severe COVID-19 and interferon therapy responsiveness	[38,39,40,41,42,43,44]
STAT3	Signal Transducer and Activator of Transcription 3	Regulator of cytokine and inflammatory signaling	STAT3 hyperactivation contributes to cytokine storm	Target for immunomodulatory therapy	[44,45,46,47,48]
SRC	SRC Proto-Oncogene, Non-Receptor Tyrosine Kinase	Immune and epithelial signaling kinase	Amplifies inflammatory lung signaling in COVID-19	Potential target to reduce lung inflammation	[49,50,51,52]
HCK	Hematopoietic Cell Kinase	Regulates macrophage and neutrophil activation	Promotes myeloid-driven inflammation in COVID-19	Target for macrophage-mediated cytokine storm control	[53,54,55]
SYK	Spleen Tyrosine Kinase	Controls Fc receptor immune signaling	Drives immune-complex-mediated lung inflammation	SYK inhibitors reduce pulmonary inflammation	[56,57,58]
EGFR	Epidermal Growth Factor Receptor	Regulates epithelial repair and survival signaling	Contributes to lung injury and fibrosis in COVID-19	Target for preventing pulmonary remodeling	[59,60,61]
PIK3CB	PI3K Catalytic Subunit Beta	Catalytic component of PI3K/AKT pathway	Supports immune metabolic reprogramming in COVID-19	Target for immunometabolic modulation	[62,63,64]
PIK3R1	PI3K Regulatory Subunit 1	Regulatory controller of PI3K activation	Dysregulated PI3K signaling in COVID-19 immunity	Biomarker of immune-metabolic imbalance	[65,66,67]
HSP90AA1	Heat Shock Protein 90 Alpha	Protein folding and viral protein stabilization	Facilitates SARS-CoV-2 replication and stress response	Antiviral therapeutic target	[68,69,70]
PTPN11	Protein Tyrosine Phosphatase Non-Receptor Type 11 (SHP2)	Regulates cytokine and checkpoint signaling	Modulates JAK/STAT and MAPK dysregulation in COVID-19	Target for restoring immune signaling balance	[71,72,73]

**Table 4:** Molecular Docking Results of PN-COVID-19 Hub Genes.

Hub Gene	Co-Crystallized Ligand Score (kcal/mol)	Best-Ranked PN Phytochemical	Phytochemical Docking Score (kcal/mol)	Comparative Interpretation
STAT1	−10.8	Procyanidin B2	−9.0	Lower binding than control, but stable interaction
STAT1	−10.8	Diosgenin	−9.0	Weaker affinity than the control, yet favorable binding
STAT1	−10.8	Gnidimacrin	−9.0	Reduced binding compared with the co-crystallized ligand
PIK3CB	−9.2	Diosgenin	−9.9	Stronger binding than the co-crystallized ligand
SYK	−8.3	Gnidicin	−9.4	Significantly stronger binding than the control
HSP90AA1	−9.8	7,7′-Dihydroxy-3,8′-biscoumarin	−10.1	Superior binding affinity compared to the control
PIK3R1	−8.2	Pimelea factor P2	−11.0	Markedly stronger binding than the co-crystallized ligand
EGFR	−8.4	Diosgenin	−9.4	Enhanced binding relative to control
SRC	−7.8	Rutin	−10.5	Substantially stronger binding than the co-crystallized ligand
HCK	−7.9	Procyanidin B2	−10.5	Significantly improved binding affinity
STAT3	−8.9	Procyanidin B2	−8.7	Comparable binding with a slight reduction
PTPN11	−7.5	Quercetin	−9.2	Stronger binding than the co-crystallized ligand

**Table 5:** Molecular Docking Results of PN Phytochemicals Against SARS-CoV-2 Main Protease (Mpro).

Ligand/Compound Name	Docking Score (kcal/mol)	Comparative Analysis of PN Phytochemicals vs. Co-Crystallized Ligand
Co-crystallized ligand (control)	−8.3	Reference compound used for comparative docking evaluation.
Oleanolic acid	−12.9	Exhibited a substantially stronger binding affinity than the co-crystallized ligand, indicating superior predicted inhibitory potential.
Epigallocatechin gallate	−9.4	Demonstrated notably stronger binding than the co-crystallized ligand, suggesting enhanced interaction with the Mpro active site.
Quercetin-3-O-β-D-(6′-galloyl)-glucopyranoside	−8.6	Showed slightly improved binding affinity compared to the co-crystallized ligand, indicating comparable inhibitory potential.
Quercetin-3-O-arabinoside	−8.4	Displayed marginally better binding than the co-crystallized ligand, suggesting similar binding stability.
Pimelea factor P2	−8.3	Exhibited binding affinity equivalent to the co-crystallized ligand, indicating comparable inhibitory strength.
Gnidicin	−8.2	Showed slightly weaker binding than the co-crystallized ligand, though still within a favorable interaction range.
Procyanidin B2	−8.2	Demonstrated slightly lower affinity than the co-crystallized ligand but maintained stable predicted binding.
2,4′,6-Trihydroxy-4-methoxybenzophenone-2-O-glucoside	−8.2	Presented marginally reduced affinity compared with the co-crystallized ligand, yet retained good binding potential.
7,7′-Dihydroxy-3,8′-biscoumarin	−8.0	Displayed lower binding affinity than the co-crystallized ligand, suggesting comparatively weaker inhibition.

**Table 6:** Molecular Docking Results of PN Phytochemicals Against SARS-CoV-2 Papain-like protease (PLpro).

Ligand/Compound Name	Docking Score (kcal/mol)	Comparative Analysis of PN Phytochemicals vs. Co-Crystallized Ligand
Co-crystallized ligand (control)	−6.4	Reference compound used for comparative docking evaluation.
Quercetin-3-O-β-D-(6′-galloyl)-glucopyranoside	−8.4	Displayed substantially stronger binding affinity than the co-crystallized ligand, indicating superior predicted inhibitory potential.
2,4′,6-Trihydroxy-4-methoxybenzophenone-2-O-glucoside	−8.3	Demonstrated markedly enhanced binding compared with the co-crystallized ligand, suggesting improved active-site interaction.
Procyanidin B2	−8.1	Exhibited significantly stronger binding affinity than the co-crystallized ligand, supporting stable inhibitory interactions.
Oleanolic acid	−7.6	Showed improved binding affinity relative to the co-crystallized ligand, indicating favorable interaction stability.
Gnidicin	−7.4	Demonstrated stronger binding than the co-crystallized ligand, suggesting enhanced inhibitory potential.
Quercetin-3-O-arabinoside	−7.4	Exhibited a stronger affinity than the co-crystallized ligand, indicating comparable binding stability.
Pimelea factor P2	−7.3	Showed improved binding compared with the co-crystallized ligand, suggesting a favorable interaction.
7,7′-Dihydroxy-3,8′-biscoumarin	−7.2	Displayed better binding affinity than the co-crystallized ligand, though slightly weaker than top-ranking phytochemicals.
Diosgenin	−7.2	Demonstrated improved binding relative to the co-crystallized ligand, indicating potential inhibitory activity.

**Table 7:** RMSD redocking validation results of co-crystallized ligands.

Target Protein	PDB ID	RMSD (Å)
STAT1	1YVL	0.125
STAT3	6NUQ	0.081
HSP90AA1	4R3M	0.072
PIK3CB	4PUZ	0.083
PIK3R1	5XGI	0.093
EGFR	4R3P	0.087
SRC	2SRC	0.116
SYK	4XG4	0.092
HCK	5H0B	0.094
PTPN11	6BN5	0.101
Mpro	8DZ2	0.077
PLpro	7CJM	0.080

**Table 8:** KEGG pathway enrichment analysis of PN-COVID-19.

Enrichment FDR	No. of Genes	Pathway Genes	Fold Enrichment	Pathways
1.1 × 10^−9^	5	71	163.8	Prolactin signaling pathway
4.0 × 10^−11^	6	90	155.1	PD-L1 expression and PD-1 checkpoint pathway in cancer
1.3 × 10^−9^	5	77	151	Pancreatic cancer
1.4 × 10^−9^	5	79	147.2	EGFR tyrosine kinase inhibitor resistance
6.5 × 10^−11^	6	104	134.2	C-type lectin receptor signaling pathway
4.0 × 10^−11^	7	194	83.9	Kaposi sarcoma-associated herpesvirus infection
9.1 × 10^−10^	6	168	83.1	JAK-STAT signaling pathway
1.1 × 10^−9^	6	181	77.1	Herpes simplex virus 1 infection
1.1 × 10^−9^	6	190	73.4	Chemokine signaling pathway
1.3 × 10^−9^	6	203	68.7	Proteoglycans in cancer

## Data Availability

The data are available upon request.

## Supplementary Materials

The following supporting information can be downloaded at: https://www.mdpi.com/article/10.3390/biomedicines14051022/s1. Figures S1–S14; Table S1: Redocked results (Supplementary Excel file); Table S2: All hub gene docking results.
