# Supplementary material for: Leveraging ADMET Profiling, Network Pharmacology, and Molecular Docking to Evaluate the Repurposing of Product Nkabinde for COVID-19 Treatment
Source: Biomedicines. 2026 Apr 30;14(5):1022. doi: 10.3390/biomedicines14051022 (PMC13203697; doi:10.3390/biomedicines14051022)
Supplement: Supplementary file 1 [file biomedicines-14-01022-s001.zip › biomedicines-4199324_supfilescombined.pdf]

biomedicines

Supplementary Materials

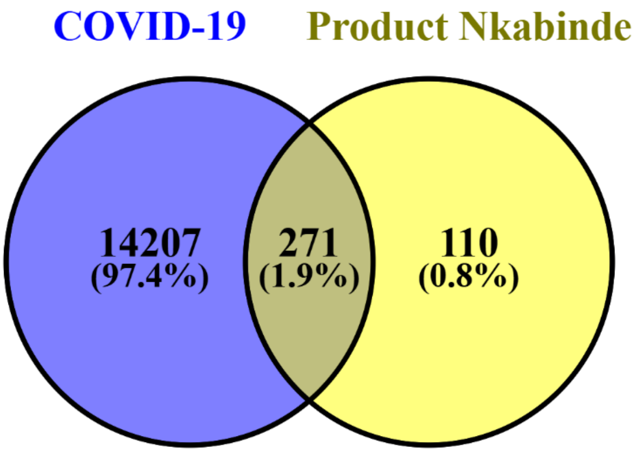

Figure S1. Venn diagram showing the overlap between COVID-19-associated host genes (14,207) and predicted molecular targets of PN phytochemicals (110), with 271 shared targets highlighting the potential multi-target therapeutic relevance of PN in COVID-19.

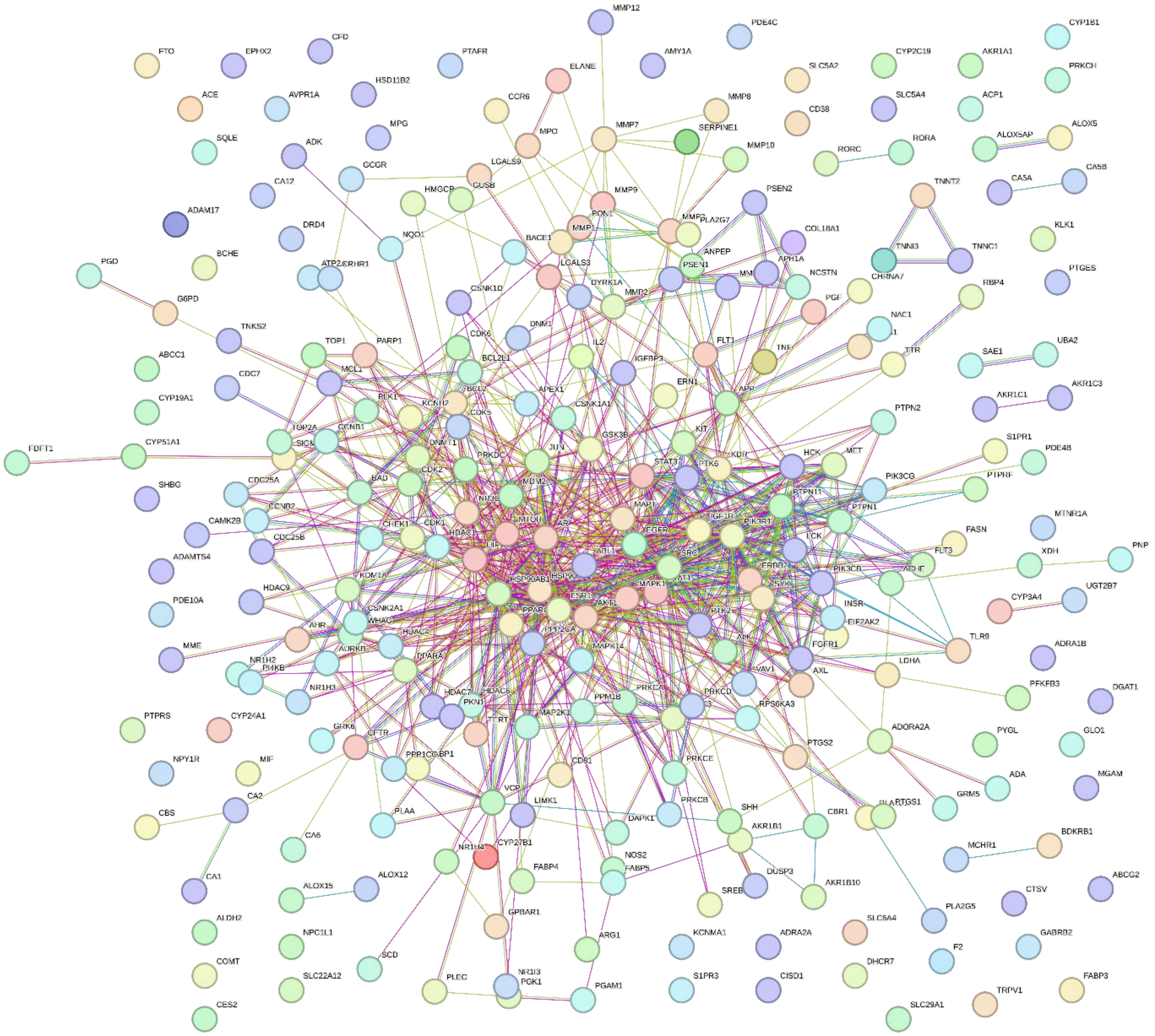

Figure S2. Protein–protein interaction (PPI) network of the 271 overlapping host targets between PN phytochemicals and COVID-19–associated genes, illustrating the complex molecular interactions underlying the potential multi-target therapeutic relevance of PN in COVID-19.

https://doi.org/10.3390/xxxxx

Supplementary Figures S3-S14: The original 2D structures of the cocrystallized ligand are in green, while the re-docked cocrystallized ligand is in magenta.

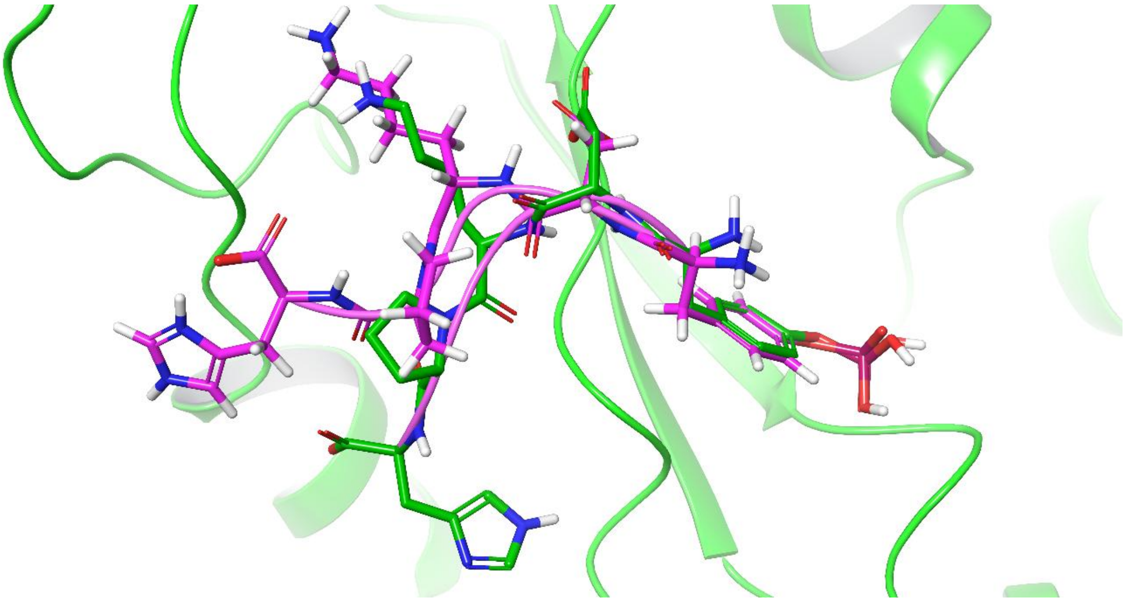

Figure S3. STAT1.

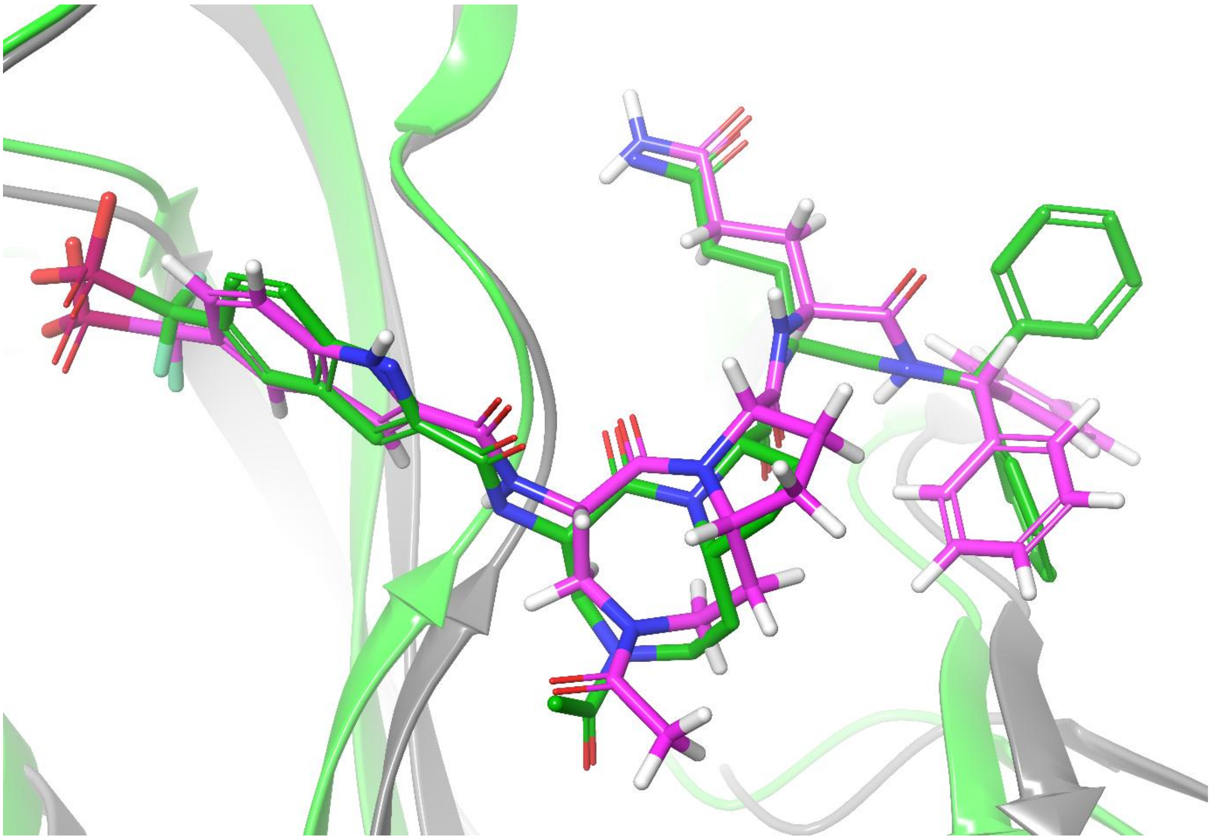

Figure S4. STAT3.

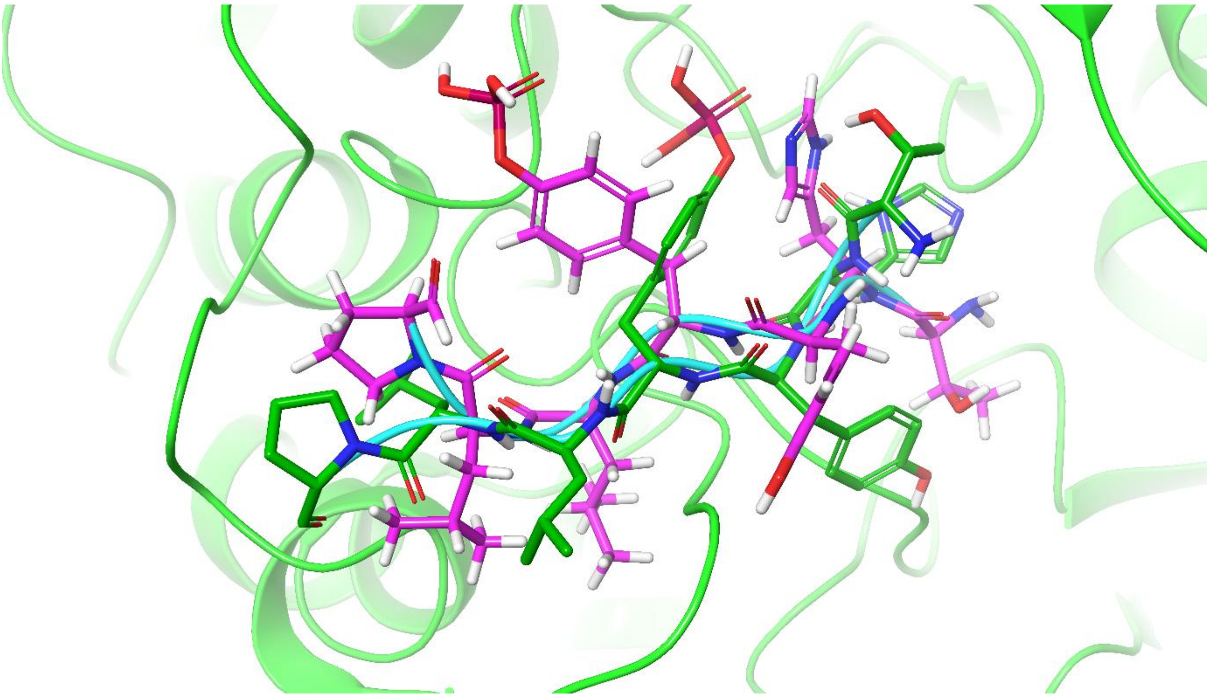

Figure S5. EGFR.

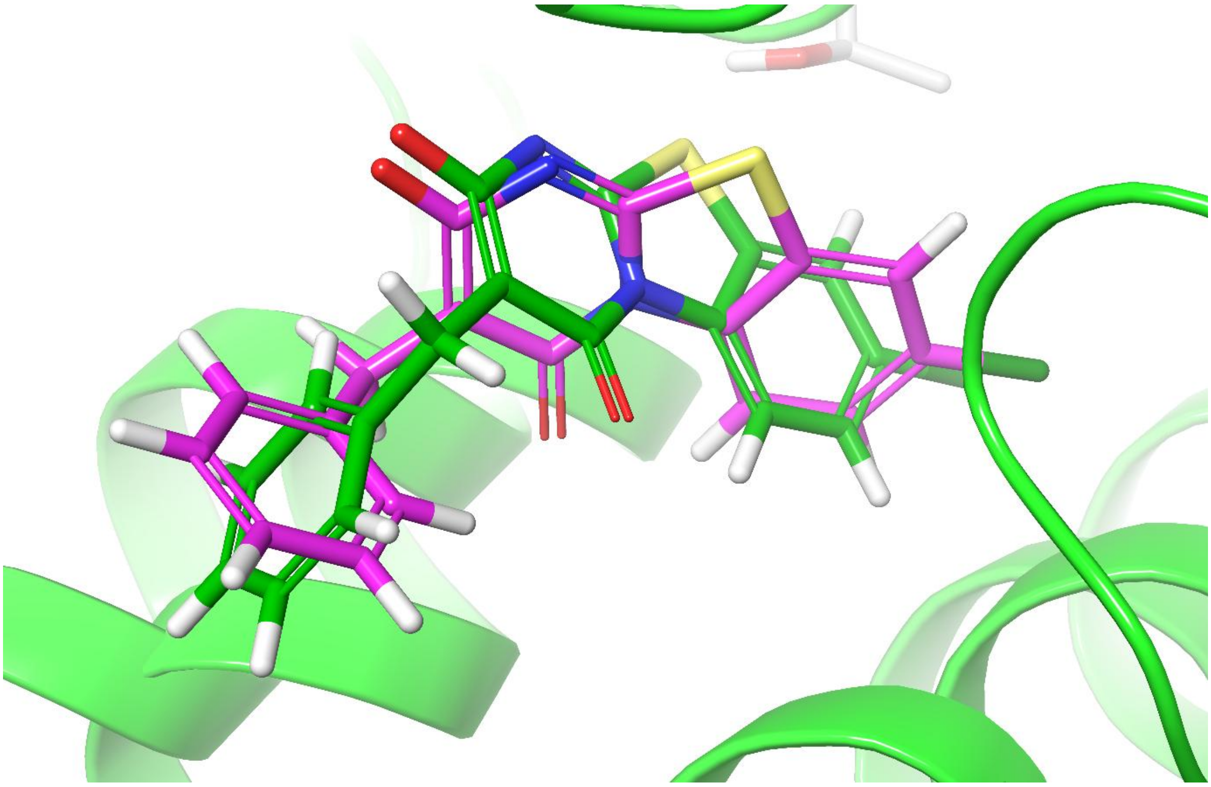

Figure S6. PTPN11.

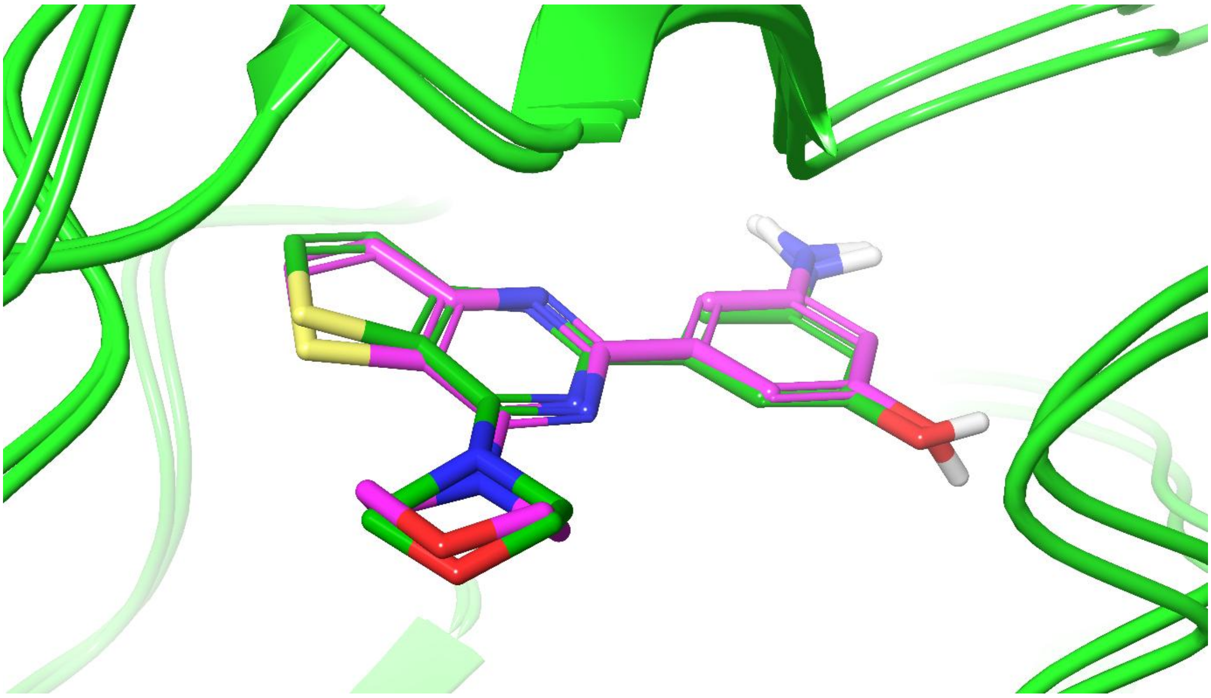

Figure S7. PIK3R1.

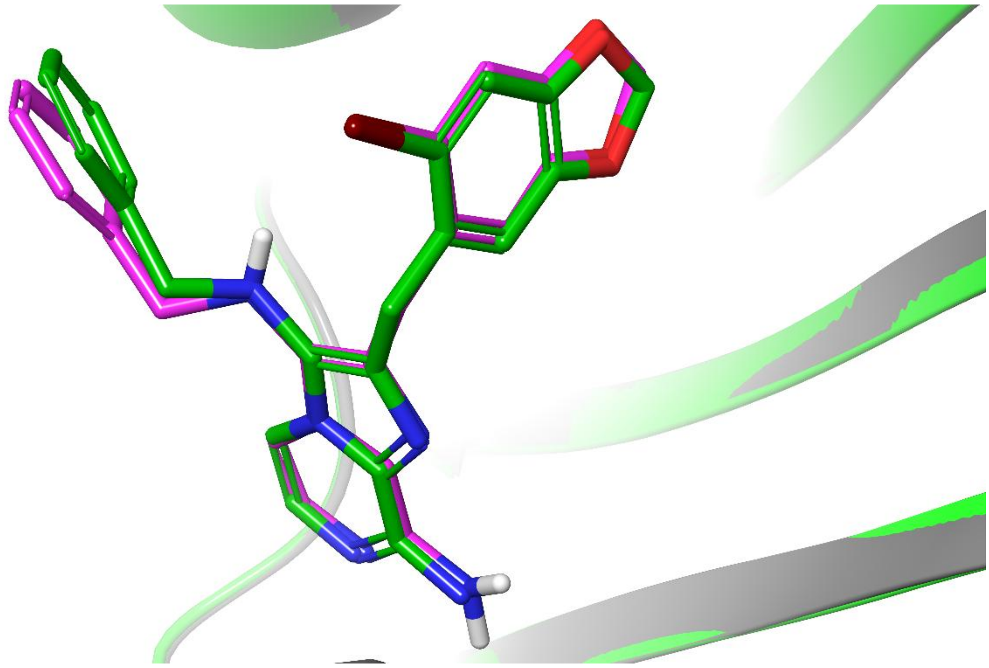

Figure S8. HSP90AA1.

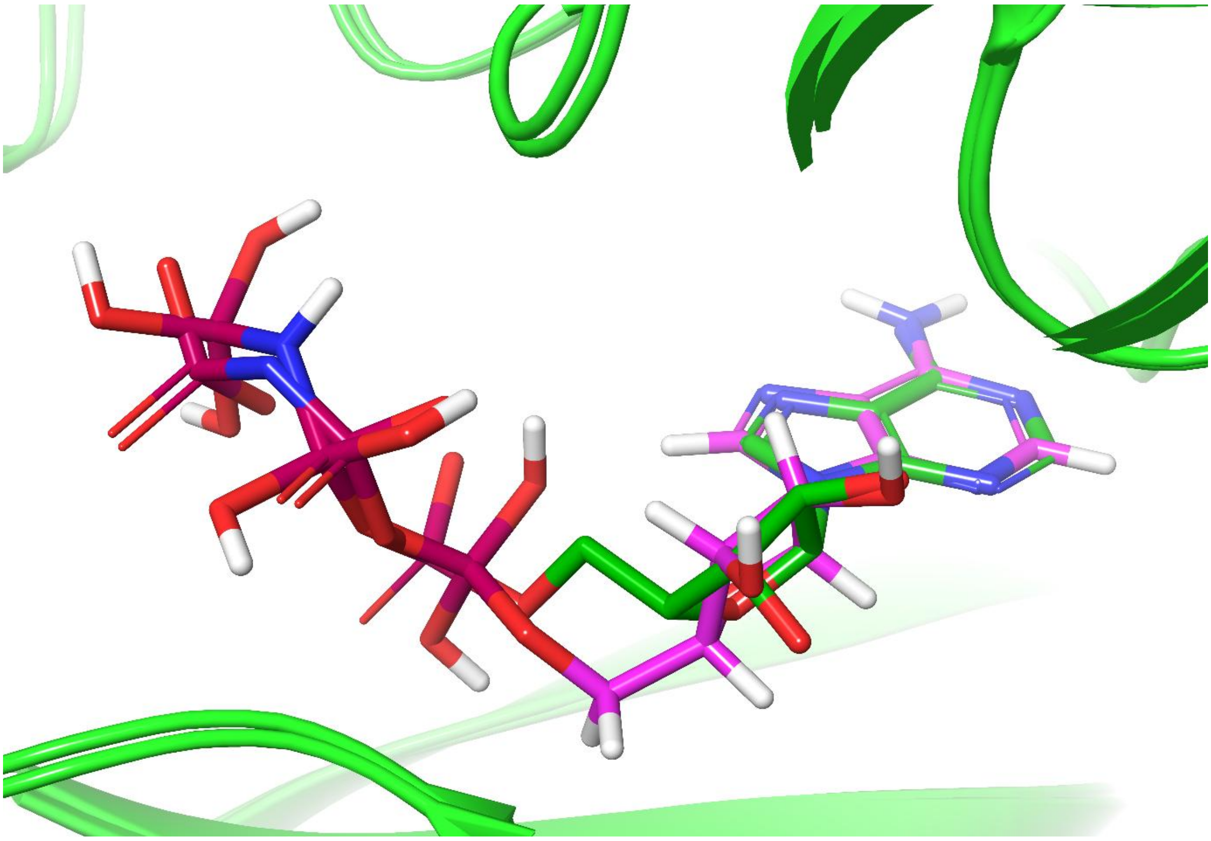

Figure S9. SRC.

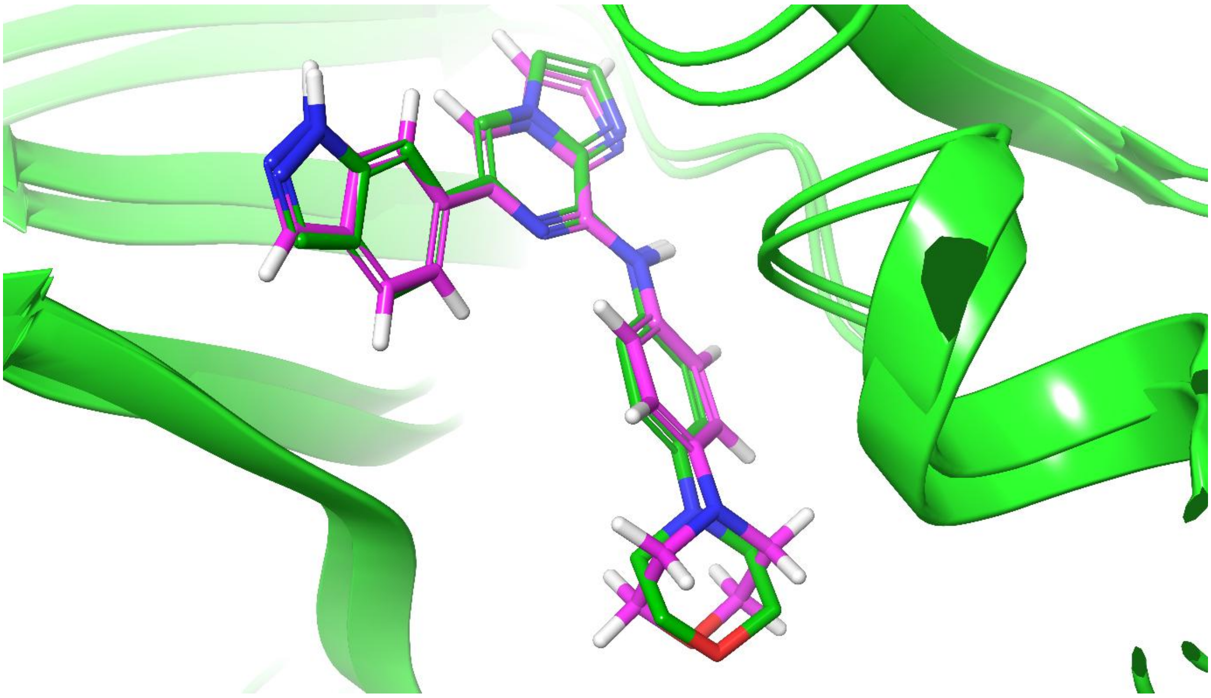

Figure S10. PIK3CB.

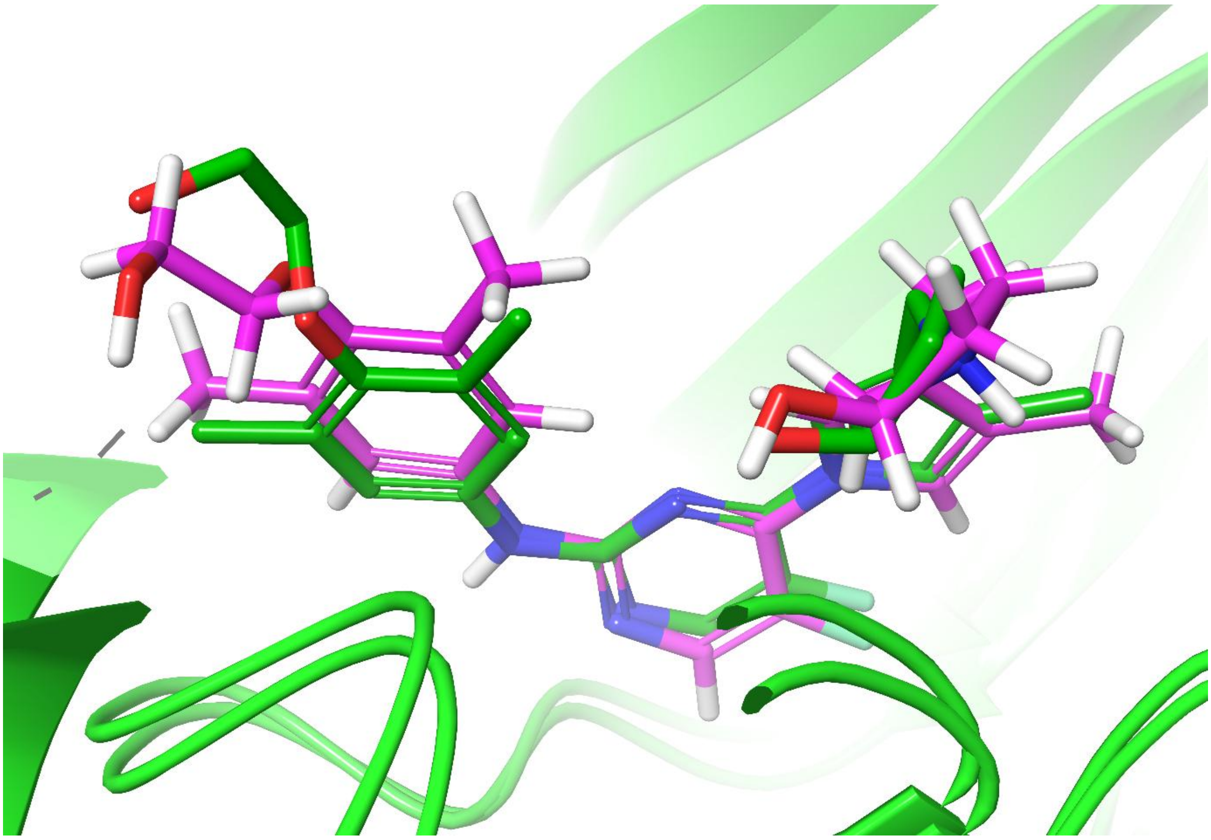

Figure S11. SYK.

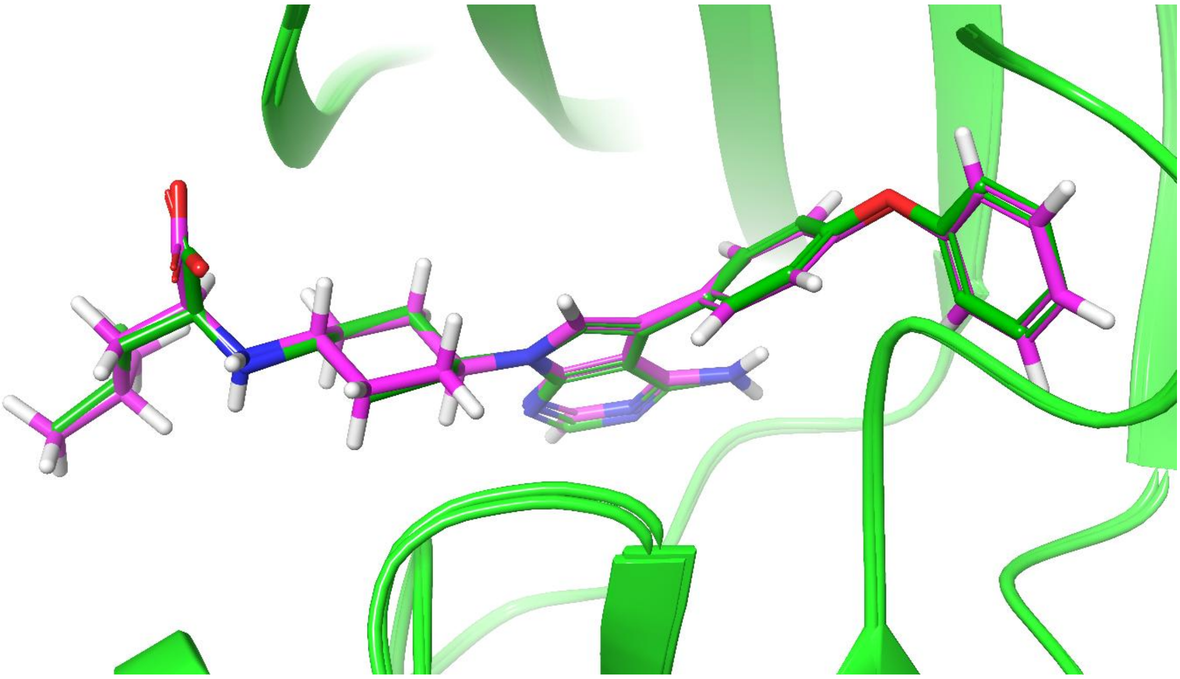

Figure 12. HCK.

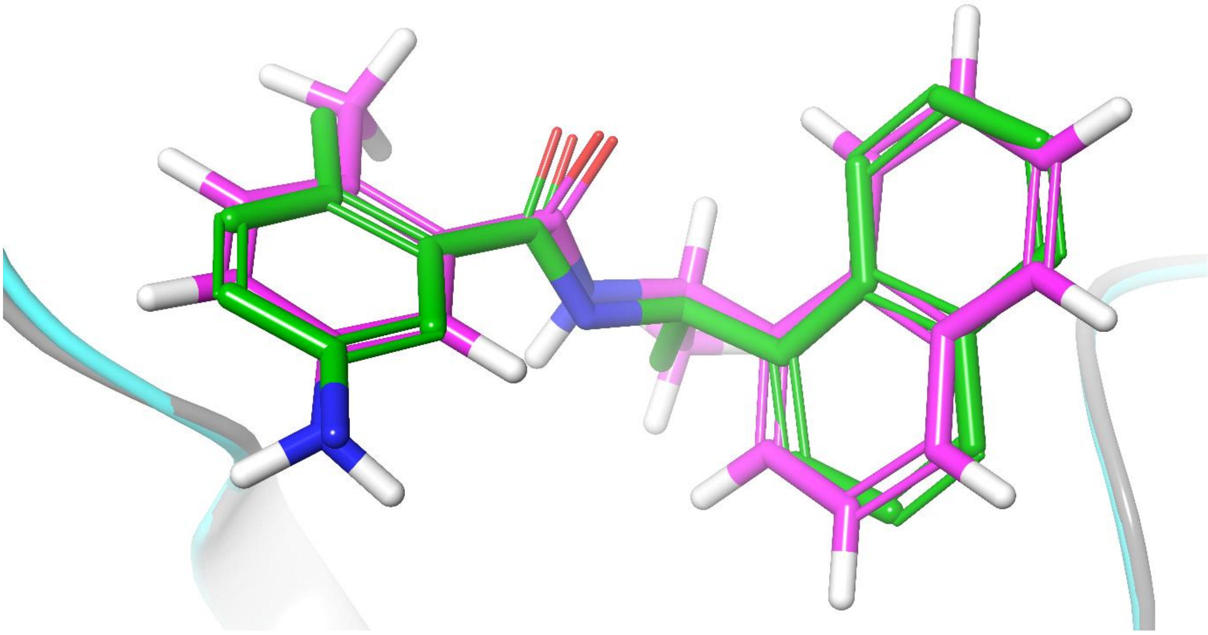

Figure S13. PLpro.

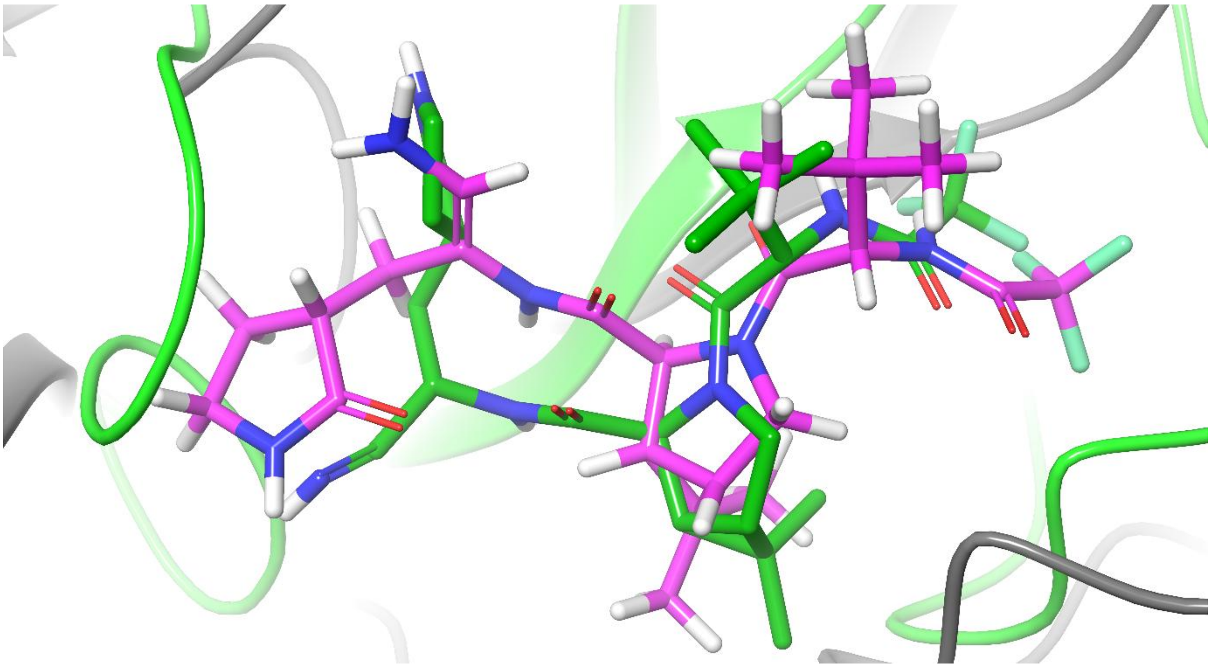

Figure 14. Mpro.

Table S1. Redocked Results (Supplementary Excel file).

PN Phytochemical	STAT1	STAT3	SRC	EGFR	PTPN11	HCK	HSP90AA1	PIK3CB	PIK3R1	SYK
quercetin-3-O-β-D-(6′-galloyl)-glucopyranoside	−8.6	−8.6	−10.6	−8.3	−8.1	−10.7	−10.5	−8.7	−10.2	−8.2
Diosgenin	−8.9	−8.6	−10.0	−9.4	−9.2	−10.6	−10.2	−9.9	−10.5	−8.7
Prostratin	−7.9	−7.6	−8.4	−7.8	−7.5	−8.1	−7.3	−7.8	−8.0	−7.6
6-(8″-Umbelliferyl)-apigenin	−8.2	−8.3	−10.6	−8.7	−8.1	−10.7	−9.4	−8.2	−10.2	−8.1
Pimelea factor P2	−8.7	−8.4	−10.3	−8.3	−8.2	−10.0	−8.3	−8.7	−11.0	−8.7
Wikstroelide A	−8.1	−7.9	−9.4	−8.0	−8.0	−9.6	−9.2	−7.9	−9.8	−7.8
Gnidicin	−8.5	−8.5	−10.2	−8.9	−8.9	−10.0	−8.2	−9.1	−10.4	−9.4
Gnidilatidin	−8.4	−8.3	−9.9	−8.5	−8.6	−9.8	−9.0	−8.1	−9.9	−8.3
Gnidimacrin	−9.0	−8.5	−10.2	−8.6	−8.6	−9.8	−7.8	−9.2	−10.1	−8.5
(−)-Epicatechin	−8.0	−7.8	−8.9	−7.9	−7.7	−9.0	−8.1	−7.9	−8.8	−7.8
Oleanolic acid	−8.8	−8.2	−10.0	−8.0	−8.9	−10.3	−9.5	−8.9	−10.3	−14.0
Procyanidin B2	−9.0	−8.7	−10.2	−8.3	−8.1	−10.5	−8.8	−8.5	−10.1	−8.4
Epigallocatechin gallate	−8.7	−8.7	−9.6	−8.1	−8.0	−9.9	−8.4	−8.2	−9.8	−8.7
Quercetin	−8.4	−8.2	−9.8	−8.0	−9.2	−9.6	−8.0	−8.1	−10.6	−8.0
Catechin	−8.1	−7.9	−9.0	−7.8	−7.7	−9.1	−7.6	−7.9	−8.9	−7.8
Emodin	−8.2	−8.0	−9.4	−7.9	−8.0	−9.3	−8.9	−7.8	−9.5	−7.9
Daucosterol	−7.9	−7.6	−8.8	−7.5	−7.6	−9.0	−7.8	−8.5	−9.2	−7.6
β-Sitosterol	−8.0	−7.7	−9.1	−7.6	−7.8	−9.2	−7.7	−14.2	−9.0	−7.7
Rutin	−8.6	−8.7	−10.5	−8.7	−8.6	−10.6	−8.1	−8.6	−10.6	−8.7
Chrysophanol	−7.8	−7.6	−8.9	−7.7	−7.6	−9.0	−8.3	−7.9	−8.9	−7.6
Physcion	−7.9	−7.7	−9.0	−7.8	−7.7	−9.1	−8.5	−7.9	−9.0	−7.7
7,7′-dihydroxy-3,8′-bis-coumarin	-8.1	-8.3	-8.2	-8.0	-9.1	-9.2	-7.6	-7.9	-9.2	-8.0
Aloin	−8.0	−7.9	−9.2	−7.9	−7.8	−9.3	−8.8	−7.9	−9.4	−8.7
Gallic acid	−6.9	−6.7	−7.8	−6.8	−6.9	−8.0	−7.2	−5.7	−7.9	−6.8
Quercetin-3-O-arabinoside	−8.3	−8.2	−9.9	−8.0	−8.1	−9.8	−8.6	−8.4	−9.9	−8.2
2,4′,6-trihydroxy-4-methoxybenzophenone-2-O-glucoside	−8.1	−7.9	−9.4	−7.8	−7.9	−9.3	−7.8	−7.9	−9.5	−7.8
2,3,4′,5,6-pentahydroxybenzophenone-4-C-glucoside	−8.0	−7.8	−9.2	−7.7	−7.8	−9.1	−8.2	−7.8	−9.3	−7.7

Table S2. All hub gene docking results.
